# Authoritarian multiparty governments

**DOI:** 10.1080/13510347.2024.2338858

**Published:** 2024-04-17

**Authors:** Laure Bokobza, Jacob Nyrup

**Affiliations:** a The Behavioural Insights Team, London, United Kingdom; b Department of Political Science, University of Oslo, Oslo, Norway

**Keywords:** political parties, autocratic politics, government, power sharing, cabinet appointments, co-optation

## Abstract

In 2020, almost 50% of dictatorships included multiple parties in their government. Existing research has not studied this systematically, and generally considers granting outsiders access to ministerial cabinets a risky and costly strategy. This article provides evidence of authoritarian multiparty governments (AMGs) being a global and increasingly common phenomena. We argue that AMGs are a power-sharing strategy whereby autocrats co-opt outsiders through cabinet appointments to divide the opposition and consolidate their rule. Using individual-level data on ministers' partisan affiliation in autocracies worldwide, we show that AMGs positively correlate with ethnic divides, civil war, and “democratic” institutions, and we use new measures of power sharing to demonstrate that coalition partners often exert a real influence through their ministerial mandates. Our findings suggest that AMG is an overlooked survival strategy for autocrats and highlight the need to focus more on the organization and composition of the political executive in autocracies.

Recent research on authoritarianism has moved away from the long-held assumption that when there is a ruling party, it governs alone. Scholars have instead underscored that autocracies may allow several parties to enter the political arena and compete in elections.^1^ Generally speaking, this allows dictators to maintain a façade of democratic institutions, while limiting access to executive power, identifying threatening opposition fringes, and targeting spoils to consolidate their rule.^2^ However, a concomitant phenomenon has been under-researched: The presence of multiple parties within the highest civilian instances of decision-making, namely ministerial cabinets. In 2020, this was true in almost half of the world's autocracies, from Rwanda to Mali, Iran, and Malaysia. We define this as authoritarian multiparty government (AMG hereafter). In this article, we set out to investigate this phenomenon on a global scale: We motivate its theoretical relevance as a distinct and thus far largely overlooked category of authoritarian regimes, explore its evolution over time and empirical correlates, and argue that it can be used as a basis for novel and fine-grained measures of authoritarian power sharing.

Autocrats adopt regime-sanctioned parties to foster stability,^3^ mitigate threats from below,^4^ manage intra-elite conflict, and solve inter-temporal power-sharing dilemmas.^5^ Dictators thus retain and consolidate power by offering rents to their supporters. Such rents often come in the form of appointments to key positions, including ministerial portfolios. Recent work has highlighted the importance of ministerial cabinets in autocracies specifically as policy-making and power-sharing institutions shaping how constrained the ruler is.^6^ In this light, it is puzzling that autocrats would appoint cabinet members from parties other than their own, since it may ultimately undermine their own rule.

Building on recent studies which suggest that ruling parties in autocracies are generally weaker institutions than we take them to be, we argue that AMGs are power-sharing arrangements between parties that represent different groups in society.^7^ These groups can be defined along, for example, pre-existing ethnic, political, or economic cleavages. Some of them decide that they are better off entering into the governing coalition than competing with each other or challenging the incumbent. We depart from the existing literature by considering the possibility that upon entering the cabinet, co-opted individuals may retain their affiliation with different parties, so that multiple parties are ruling together. This arrangement is beneficial for autocrats: By co-opting a select part of the opposition without subsuming it under the ruling party's umbrella, they prevent a strong, unified opposition from emerging.^8^ It is also advantageous for the coalition parties, who benefit from the financial and non-financial perks of being in office without compromising their status as distinct from the ruling party. Both sides are also better off than under full democracy, where they risk losing control of government to outsider parties. AMGs should therefore be considered as stable autocratic equilibrium, rather than a steppingstone to democracy.^9^

Importantly, these power-sharing arrangements are not all the same: They vary in the extent to which power is shared and coalition parties exert real influence over policy-making. At one end of the continuum, AMGs can be *pro-forma* arrangements, in which the ruler fully controls his or her coalition partners. One could expect the majority of AMGs to be arrangements in which rulers only ever engage in “for the show.”

To the contrary, we argue that many of these governments exhibit features of what we call *bona fide* power sharing, where the coalition partners are separate entities with independent party structures and their own political agenda. Granting cabinet positions to other parties then contributes to the ruler’s survival strategy. These appointments serve as buy-offs, entrenching the co-opted opposition in dependence towards the ruling party for resources, and preventing the emergence of a united opposition front. Meanwhile, co-opted parties get ministerial mandates and a say in policy-making, while benefiting from the spoils of being in office.

We do not expect AMGs to be randomly distributed among the pool of autocracies. Instead, they should be particularly likely to emerge where there are strong and salient underlying societal divides, such as those inherited from past civil wars or a history of social and cultural cleavages, which make it more difficult for the ruler to outright overtake the ruling coalition. Power sharing is also more likely where there are other nominally democratic institutions in place, allowing members of coalition parties to organize and compete outside of the ruling coalition, and creating a persistent threat to the incumbent.

We proceed to explore the rise of AMGs empirically. To do so, we rely on a recent dataset on government composition, WhoGov.^10^ WhoGov contains individual-level data, including party affiliation, for cabinet members globally in the period 1966–2020.

We exploit this incomparably rich data and show first that multiparty government has become increasingly common among authoritarian countries. In 2020, almost 50% of all autocracies had more than one party in government, against less than 25% forty years earlier. Furthermore, we show that AMGs are conceptually distinct from democracies, and are no more likely to democratize than autocracies without AMG.

Next, we explore which autocratic regimes have adopted AMGs. We show that, in line with our theoretical expectations, the phenomenon correlates positively with social and cultural cleavages, prior civil wars, as well as with the presence of nominally democratic institutions. The fact that the AMGs correlate with stark societal divides which make it harder for the ruling party to overtake coalition partners, and with the presence of other institutions making the threat of opposition coalescing more credible, suggests that there is real power sharing.

At last, we investigate the internal dynamics of AMGs, and show that while the dictator's party is almost always the most powerful in terms of share and importance of cabinet positions, there are in many cases signs of real power sharing between the autocrat's party and the coalition parties. In particular, we build a Herfindahl-Hirschman Index of party concentration in government, weighted by the importance of ministerial portfolios. The index indicates the level of government party control, ranging from 0 (the governing party has no cabinet positions) to 1 (the governing party controls all cabinet positions). Results indicate that *bona fide* multiparty governments are surprisingly common among AMGs.

The article advances the study of contemporary authoritarian regimes in several ways. First, we show that modern authoritarian regimes do not only adopt democratic institutions such as multiparty elections and parliaments but surprisingly often share the spoils of executive power with other parties through cabinet appointments. Based on this, we categorize autocracies into AMGs and non-AMGs, and provide a theoretical motivation for why this distinction is important.

Our second main contribution is the use of individual-level data on the composition of cabinets in autocracies around the world. This allows for a fine-grained *and* global analysis of co-optation and intra-elite dynamics in authoritarian regimes. Taken together, our results – while explorative – suggest that the field would benefit from adopting a broader and more nuanced perspective on the role of parties – rather than *the party* – in the political executive.

## Political parties and authoritarian rule

Political parties are viewed as one of the pillars of modern authoritarian rule.^11^ The so-called “third wave of democratization” also saw the rise of autocracies organized politically and institutionally around the existence of a ruling party.^12^ Originally, these regimes were often referred to as “single-party” states – when no party other than that sanctioned by the regime is allowed^13^ – or “dominant-party” regimes – meaning that other parties can legally compete to various degrees through elections in a “competitive authoritarian” system.^14^

More recently, scholars have gone further in distinguishing between different kinds of authoritarian party-based regimes, looking at constitutional rules, the composition of the legislature, and the holding of competitive elections. Hadenius and Teorell^15^ and Magaloni, Chu and Min^16^ use “multiparty regimes” to designate those cases in which the ruling party allows the creation by opposition groups of parties who participate in elections and the legislature. This definition, which puts the emphasis on formal rules and electoral competition, is close theoretically to that of “electoral democracies”^17^ or “competitive authoritarian” regimes.^18^ Still, these studies have in common that they view the ruling party as the focal point of the political life of the regime.

Consequently, research has strived to understand why dictators create regime-sanctioned political parties. Ruling authoritarian parties have been shown to serve intertwined roles which ultimately foster better regime outcomes, including durability^19^ and resilience to internal challenges such as coups and insurgencies.^20^ Their first function is to mobilize mass support.^21^ The party machine can be used as a tool to control the masses and gather information, but also to foment support and entrench the dependence of the masses, who develop an interest in the regime's survival.^22^

### Co-optation dynamics and ruling parties in autocracies

Authoritarian parties' second function is one of bargaining, co-optation, and management of threats coming from intra-elite conflicts. Part of the literature has focused on the granting of spoils *within* the ruler's own support coalition to enhance elite cohesion and deter potential challengers.

A related strand of research studies how institutions such as legislatures and elections allow the ruler to co-opt elites beyond his or her own party.^23^ Through institutional rules, the ruler manages the structural relationships between the state and political opponents, as well as “among various opposition groups themselves.”^24^ This has consequences downstream on the level of opposition to regime policies,^25^ and on leadership succession and regime transitions.^26^

Indeed, the maintenance of intra-elite cohesion goes hand in hand with the channelling of opposition demands, with the aim of preventing any strong, charismatic opposition figure from emerging. In the words of O'Donnell,^27^ through co-optation, autocrats “encapsulat[e]” some sectors of the opposition to maintain themselves in power (cited by Gandhi and Przeworski^28^). Frantz and Kendall-Taylor^29^ further argue that allowing for multiple opposition parties to exist and participate in elections is a rational co-optation strategy on the ruler's part, “enabling a wider range of the opposition to choose the degree to which they wish to associate with the regime.”

Nonetheless, this co-optation logic often works to the ruler's advantage. Malesky and Schuler^30^ show that the policy influence of potential opposition figures through the legislature remains limited and strictly defined by parliamentary rules set by the regime. Relatedly, Reuter and Robertson^31^ use data from Russia to show how authoritarian legislatures can help reduce anti-regime protests by providing rent-seeking opportunities to key opposition elites. Specifically, they claim that legislatures allow for “personal co-optation” in the sense that they enable the ruler to direct particularistic benefits to well-identified potential challengers. Elections and legislatures are not the only institutions through which the co-optation of opposition elites operates. In this article, we argue that the targeting of perks also works through cabinet appointments.

### Shifting the focus to ministerial cabinets

Studies of authoritarian rule point to several reasons why autocrats may choose to include selected parts of the opposition in their cabinet. One answer may lie in the fact that authoritarian ruling parties are rarely as strong as we take them to be.^32^ Leaders of parties that lack the key condition for institutional strength, namely organizational autonomy, may find themselves pressured into incorporating part of the opposition into the ruling coalition. Institutions such as ruling parties can be used by the autocrat to selectively co-opt potential rivals by distributing rents. Scholars of African politics have emphasized that the prevalence of patronage politics can result in a broadening of the winning coalition beyond party lines to facilitate the distribution of rents.^33^ However, this strategy can easily turn against the ruler, as rivals can use these transfers to organize collectively against him/her, especially if he/she lacks means of exerting formal control over them. This makes this approach both costly and risky for the ruler.

Others have suggested that the co-optation of select outsider fringes can facilitate bargaining between the ruler and the opposition in the form of targeted policy concessions.^34^ This presents the added advantage of increasing coordination costs among the leader's opponents.^35^ Once a former opposition group has been subsumed into the leader's party, it makes it harder for the rest of the opposition to unite. In this vein, Arriola, Devaro and Meng^36^ demonstrate that presidential incumbents can strategically use cabinet appointments with the two-fold aim of buying off rivals *and* inducing opposition fragmentation. Using data on African dictatorships, they find that co-opted opposition figures are subsequently more likely to run as independents in the following presidential elections. This “divide-and-conquer” strategy satisfies the ruler's key objective: Preventing the emergence of a strong, unified opposition under a single-party umbrella.^37^ Following Arriola, Devaro and Meng's^38^ logic, once included in government, these individuals no longer identify with opposition parties: They relinquish their label to benefit fully from the spoils of being in the cabinet. In this view, it follows that power sharing between different parties in government should remain virtually non-existent if the ruler is successful in his or her selective co-optation strategy. How can we then explain the prevalence and persistence of autocratic governments in which several distinct parties are represented?

We build on this literature yet depart from existing works by considering the case of governments in which co-opted oppositions fringes retain their respective party labels and envisaging this as a viable survival strategy for both the leader's party and its coalition members. From the ruler's perspective, allowing outsiders to keep their party label and some independence hinders the opposition from uniting by creating a third way within autocratic politics. Political actors can choose to be affiliated to the ruling party, the opposition outside of government, or the opposition in government. This, in turn, raises the bar of how difficult it is for non-regime parties to coalesce and unite against the leader's party^39^; and buys the ruler some time to adjust his or her survival strategy to opposition threats. From the perspective of opposition parties, entering the government is a two-edged sword. On the one hand, retaining their party label ensures that they are not fully conflated with the ruling party, while being in government allows them to gain access to resources and power they would otherwise not benefit from.^40^ On the other hand, this can be seen as a betrayal from the rest of the opposition and make it harder for them to maintain a credible image as regime challengers. In the following section, we flesh out the concept of AMG theoretically.

## (Re-)thinking about multiple parties in autocracies

The presence of multiple parties in government does not imply that power is in practice shared between different groups with an effective say in policy-making, especially since the regime controls the rules of the game.^41^ *A priori*, one can think of AMGs as ranging along a continuum which goes from governments where each included party has its agenda and gets an actual say in policy-making (*bona fide* AMG), to cabinets in which the inclusion of several parties is done in a purely perfunctory manner, and does not entail actual power sharing between groups of different ideological, political, or economic strands (*pro-forma* AMG).

Because of the costs and risks associated with out-party co-optation, one may expect rational autocrats to refrain from granting the opposition in government any meaningful role. *Pro-forma* multiparty governments are characterized by the total dominance of the leader's party. Typically, either the coalition parties are satellite organizations under the dictator's control to begin with or the ruler gradually manages to take over his or her coalition partners and fully control them. This offers several advantages for the incumbent: By granting a veneer of democratic institutions to the regime, it limits the reputational, diplomatic, and economic costs associated with being an authoritarian one-party state. At the same time, these appointments foster the fragmentation of the opposition.^42^ Meanwhile, the parties that chose to enter the government coalition do so to enjoy the spoils of being in power but ultimately have little say in actual decision-making. In these cases, the real opposition parties are typically banned. Examples of such systems include former communist countries such as Poland from 1947 to 1989.

However, granting of cabinet positions to members of other parties can also serve as a strategy on the ruler's part to consolidate his or her rule by sharing power – to the least possible extent necessary. Counter-intuitively, we expect most cases of autocratic multiparty governments to not be purely *pro-forma*. This is because out-party cabinet appointments are costly for the leader. Some of these costs are direct: the neo-patrimonial authoritarianism literature suggests, cabinet appointments serve as buy-offs.^43^ They entrench newly co-opted opposition members into economic dependence by offering them more than they would get through any outside option. Moreover, cabinet posts are high-level political positions. Therefore, they come with a political clout and public visibility, which are non-monetary rewards that work towards fostering dependence on the incumbent. Crucially, *some* cabinet positions come with an important role in decision-making (e.g. Minister of Defense or Minister of Finance), so that allocating them to outsiders entails a higher degree of trust and power sharing than less consequential portfolios (e.g. Minister of Culture). By granting important cabinet positions to members of other parties, the ruler works towards preventing a strong and unified opposition from emerging and posing a serious challenge to the regime.^44^

There are also indirect costs for the ruler, related to the potential loss of revenue and power for the incumbent. As Arriola, Devaro and Meng^45^ note, “offering a cabinet position to an opposition candidate is also costly for the incumbent because it prevents him from being able to offer that position to someone from his own party or existing ruling coalition.” In other words, the ruler faces a trade-off between within-party and out-party co-optation. Furthermore, it is also risky for the autocrat: Non-co-partisan individuals do not necessarily share the ruler's ideological and political agenda, nor do they display the obvious sign of loyalty that is affiliation to the ruling party.^46^ Ministerial portfolios come with access to information and resources that newly co-opted members can decide to use to overhaul the ruler if a better opportunity arises. Precisely because of the costs and risk associated and due to the high uncertainty and secrecy that characterizes authoritarian governments, we expect that rulers would not engage in out-party cabinet co-optation unless power sharing with other parties is optimal for their survival.

Importantly, not all ruling parties are equally strong and autonomous – even in the case of single-party cabinets. They may to varying degrees rely on other institutions and/or other political actors for their economic or military security in the short- or medium-run. These external actors may not depend directly on the ruling party for their survival because they have independent sources of income and military forces of their own, or a strong grip over a given portion of the territory. This could make the benefit of co-opting these entities outweigh the cost and risks associated with out-party co-optation at a given point in time.

From the perspective of opposition parties, entering the cabinet while retaining their label can be more beneficial than joining the ruling party. Indeed, it allows them to gain access to resources and power, while maintaining a degree of nominal and organizational independence. As Frantz and Kendall-Taylor^47^ argue, they are attracted by “the lure of a greater (albeit limited) policy influence and a means of advancing their political careers.” Entering government without disowning their party label also maintains their public image as a viable alternative, distinct from the regime-sanctioned party, while enhancing their credibility as political organizations qualified to (partly) hold the reins of government. Retaining their own party label thus prevents opposition parties from being alienated by their activist base when they enter government, which Buckles (2019) shows is one of the main risks that co-opted opposition parties face. In addition, it increases their party's bargaining power in future elections and negotiations, since they maintain an independent party structure, dealing them a stronger hand than if they had merged with the ruling party, or abandoned their labels.

When some outsider parties manage to get an actual voice in government and their party organization has not been taken over outright by the ruler's party, there is what we call *bona fide* power sharing between the ruling party and coalition parties. A condition which makes this sort of governing arrangement more likely, following Magaloni and Kricheli^48^ is that “non-regime sponsored parties must be able to retain some means to threaten the dictator’s survival when he reneges on his promises by, for example, mobilizing voters in great numbers to the streets.” When such is the case, the threat of the opposition coalescing persists – for instance because semi-competitive elections allow for some opposition parties to mobilize voters and form alliances against the ruling party. In this scenario, elites can take advantage of the incumbent's weakness.^49^

The leader is then pressured into sharing power “in good faith,” which translates into the allocation of cabinet positions to co-opted parties. This argument follows recent research emphasizing that ruling parties are not as strong as we typically pit them to be.^50^ The higher the share of non-ruling parties in government and the more important the cabinet posts they are granted, the deeper power sharing is. *Bona fide* multiparty governments can be the result of tacit deals between parties representing specific groups in society whose interests and values are opposed, but who agree to share the spoils of government or unite against a common enemy in order not to loose their grip on power. Alternatively, it can result from the failure of the opposition to unite and form a united front, which the ruler exploits by selectively including some opposition factions in the government. Importantly, upon entering the cabinet, these groups often retain their affiliation with different parties and some independence – for the above-mentioned reasons – so that multiple parties are *de facto* ruling together.

Ultimately, we expect that not all multiparty government systems entail the same level of power sharing. While we can never know for sure what goes on behind closed doors in ministerial cabinets, *pro-forma* and *bona fide* AMGs are useful concepts to help us think through how power sharing works in practice at the government level in autocracies, and how political parties are functional vehicles allowing the identification and co-optation of potential challengers, while sending a public message of openness (to the public) and of strength (to non-co-opted members of the opposition). Importantly, we highlight that multiparty government in autocracies can in fact reflect *bona fide* power sharing between the ruling party and opposition parties.

### Correlates of AMGs

The above arguments suggest that AMGs should not emerge randomly among autocracies and point to several factors that may foster the rise of opposition groups which the autocrat will want to co-opt beyond partisan lines. We outline three factors.

First, we expect that social and cultural cleavages should contribute to power sharing across party lines.^51^ Indeed, the allocation of power across party lines is not independent from the composition of each party's respective base. Since extending cabinet appointments outside of the ruling party aims at ensuring the support of segments of society needed for regime survival, we expect the existence of strong underlying social and cultural cleavages – typically along linguistic and/or ethno-religious lines – to make it easier to identify and reward specific support groups by granting their representatives ministerial portfolios.

Second, cleavages are sometimes known to result in civil war.^52^ The aftermath of a civil war is also more likely to see governments including former opposing factions – or at least a diversity of interest groups – emerge as institutional compromises aimed at ensuring regime stability. One obvious example here, discussed at length later on, is Lebanon. Ishiyama and Widmeier^53^ show that the electoral performance of former rebel political parties is partly determined by their level of control over specific constituencies during the war. We extend this argument and expect that where rebel groups successfully transform into political parties post-conflict – and in particular where rebel-to-party clauses are included in internally sponsored peace agreements^54^ – this should favour post-war governments which include several parties.

Third, we expect AMGs to be positively associated with nominally democratic institutions in autocracies, such as legislatures and/or the holding of elections. Even though these institutions do not guarantee democracy in practice, they allow coalition parties to organize and compete outside of the ruling coalition,^55^ and “provide[s] some means for advancement into political office and limited policy influence” for them.^56^ In addition, elections are focal points that expand opportunities for mobilization and lessen constraints on collective action amongst regime opponents.^57^ This has the added effect of creating a threat to the incumbent, which in turn makes genuine power sharing the best strategy for the ruler. Because not all outside parties can be co-opted, as Buckles^58^ shows, the collusion between governmental and parts of the nongovernmental opposition serves the purpose of dismembering and fracturing the opposition by making it harder for its various factions to coordinate. For the incumbent, this constitutes a cheaper, though effective, alternative to repression.^59^

## Measuring multiparty government in autocracies

We use WhoGov to investigate the rise of multiparty government in autocracies.^60^ The dataset contains information on cabinet members in 177 countries in the period 1966–2020, including most autocracies. The data contains individual-level information including the name and gender of ministers, as well as their portfolio and – crucially – party affiliation. In Appendix I, we discuss the procedure for gathering data on party affiliation and potential issues when gathering the data. Moreover, we also conducted an extensive doublechecking of the partisan composition of all governments classified as AMGs in 1970, 1980, 1990, 2000, 2010 and 2016 respectively.^61^

For each year and for every country, we create (1) a binary measure of whether more than one party is represented in the cabinet, (2) a variable measuring the number of parties included in the cabinet, and (3) the share of government posts allocated to members of coalition parties.

To measure the depth of power sharing, we construct (4) a measure of concentration of power in government using a weighted Herfindahl–Hirschman Index (H-H). The index ranges from 0 to 1, where 1 indicates that one party that controls all “points” – or all cabinet positions at once. By contrast, a low score on this scale indicates that many parties control a few portfolios each. The weighting is such that more important posts receiving a higher weight, and accounts for the fact that not all ministerial posts entail the same prestige and responsibilities. To assign weights, we create a new measure of importance. We do so by dividing all cabinet positions into three categories (high, medium, and low) following Nyrup and Bramwell.^62^ We then assign a score of 3 to high-prestige positions, a score of 2 to medium-prestige positions, and a score of 1 to low-prestige positions. For more details on the construction of the index, see Appendix A and Appendix C.^63^

To distinguish autocracies from democracies we rely on the binary measure provided by Boix, Miller and Rosato.^64^ The latter classify a country as democratic if key executive offices are filled via free and fair elections, with at least half of all men enfranchised. This relatively minimalistic definition presents a twofold advantage. First, it does not conflate aspects of the party-system or government with features of democracy itself; and second, contrary to the DD-index,^65^ this measure does not require a peaceful transition of power for a country to be classified as democratic. Thereby, we avoid including democratic multiparty governments where one dominant party has remained in power since democratization. In Appendix D, we replicate our findings using the DD-index.^66^ Further information on the variables mentioned here and other variables used throughout the analyses are included in Appendix A.

## The rise of AMGs

**Figure 1.**
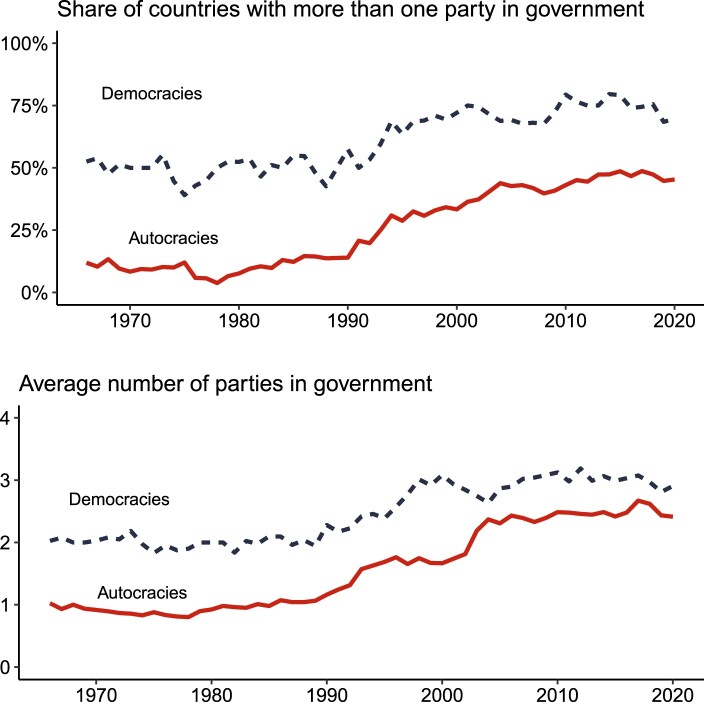
Evolution of (a) the share of countries with more than one governing party and (b) the number of parties in government.

Figure 1 shows the rise of AMGs. The top panel shows that the share of countries with more than one party in government has risen sharply and consistently in autocracies. In the 1960s and 1970s, multiparty governments were a rarity in the autocratic world (12% in 1966). By contrast, after the end of the Cold War, AMGs became increasingly common: In 2020, they constituted around 50% of all autocracies. Over the same period, we see a slight increase in the number of democracies with multiple parties in government (from 53% in 1966 to 69% in 2016).

Unsurprisingly, we find a similar pattern when looking at the average number of parties in government in the middle panel of Figure 1. Autocracies, on average, had around one party represented in government before the end of the Cold War. After the fall of the Soviet Union, the number of parties went up dramatically, and today autocracies on average have 2.4 parties in government.

Taken together, these patterns show first that AMGs have become increasingly common since the 1970s, and especially so after the end of the Cold War. Second, this evolution seems to mirror that of democracies, but the latter see a slower increase in both the number of parties represented in government and the share of ministers from coalition parties in government.

## Multi-party government, but still autocratic

One concern is that what we define as AMGs could in fact be either misclassified democracies, or regimes bound to break down and become democracies shortly after they have come into existence. If such were the case, the phenomenon we identify would not be a distinct type of power-sharing arrangement through which authoritarian leaders manage to consolidate their hold on power, but rather a form of authoritarianism in decline, or (flawed) democracy by another name.

**Figure 2.**
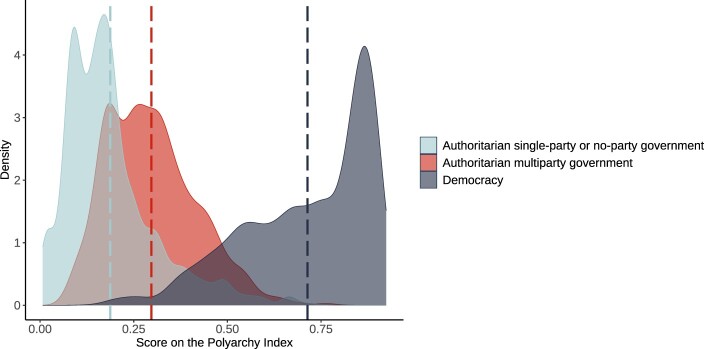
Distribution of Polyarchy scores by regime type. The vertical, dashed lines indicate the mean for the group of the same colour.

In Figure 2, we split all regimes into democracies and autocracies, and then we split autocracies into AMGs and non-AMGs. The figures show how these types of regimes are distributed along the Polyarchy score from V-Dem^67^ which captures the core “institutional guarantees” identified in Dahl,^68^ and thus can be considered as a more fine-grained measure of the level of democracy. We see that there is little overlap in the Polyarchy scores of AMGs and democracies, and it is clear from the plot that AMGs are distinct from democracies when focusing on the quality of democracy. A related concern may be that AMGs are democracies transitioning to autocracy, causing us to capture a process of democratic backsliding rather than autocratic transformation. However, out of the 1,039 country-year observations classified as AMG, only 91 (or 8%) were democratic at some point in the five years preceding their observation as AMG, meaning that democratic backsliding also cannot explain our findings.

**Figure 3.**
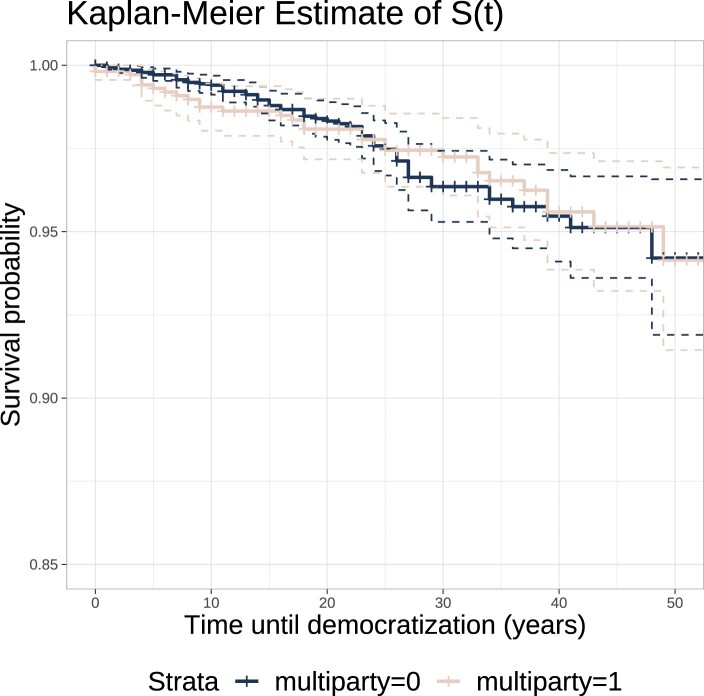
Survival probability of AMGs and non-AMGs over time.

Turning to the fate of these regimes, research has shown that the relationship between elections under authoritarianism and regime survival is complex, and that the type of elections matters.^69^ Knutsen, Nygård and Wig^70^ demonstrate the existence of a negative short-term effect, but a positive long-run impact, while Donno^71^ establishes a moderating effect of domestic and international pressures on the relationship between competitive authoritarian elections and democratization. With these findings in mind, we compare the autocratic survival prospects of AMGs and non-AMGs. We conduct a survival analysis where we model the time to democratization (which is the “event”). First, we estimate the survival probability with the Kaplan-Meier method, by AMG status and over time. Results are presented in Figure 3, with 95% confidence intervals displayed.

We proceed to test whether survival probabilities are significantly different between AMGs and non-AMGs. We run a log-rank test, with the null hypothesis being that there is no difference in survival between the two groups. This test is non-parametric, and thus makes no assumption regarding the survival distributions. The *p*-value of the test is approaching 1, which implies that there essentially is no difference in survival between the two groups. In other words, AMGs are not statistically more at risk of transitioning to democracy than their non-AMG autocratic counterparts, which is in line with Lust-Okar's^72^ finding that political liberalization is not inherently unstable. This runs counter to the claim that AMGs are merely dictatorships in decline and provides further credence to our argument that they are distinct, common, and rational power-sharing arrangements in autocracies.

## Which countries adopt AMG?

**Figure 4.**
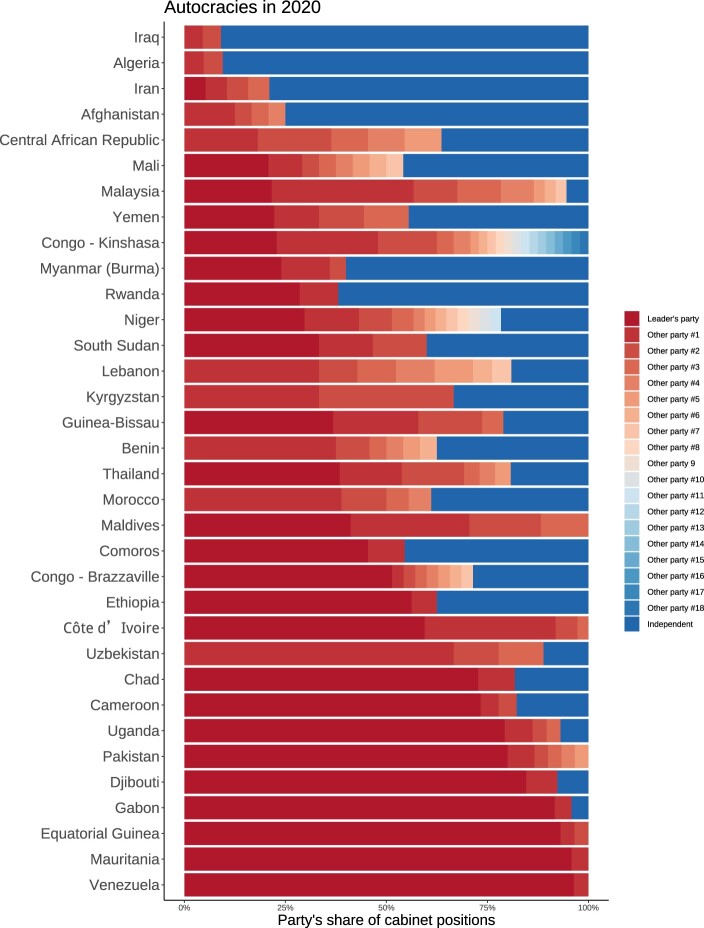
Distribution of cabinet positions by party in AMGs ranked by the size of the largest party in 2020.

Before turning to the analysis of the empirical correlates of AMGs, we provide an oversight of AMGs in 2020 in Figure 4.^73^ The figure shows the distribution of cabinet positions by parties. Countries are ranked by the share of portfolios belonging to the largest party, starting with the smallest. For instance, Congo (Kinshasa), while being classified as autocratic, has a fragile governing coalition consisting of no less than 19 different parties that each provide the support of a different section of society. At the bottom of the figure is Venezuela. Here, all ministerial positions but one belonged to the leader’s party – United Socialist Party of Venezuela. The vice president, Delcy Rodrígues, belongs to the government-aligned *Movement We Are Venezuela*. It should be noted that in some autocracies, the ruler is independent, yet one or more parties are represented in the cabinet.^74^

Are all authoritarian regimes equally likely to integrate several political parties into government? In Table 1, we correlate AMG with the three factors outlined previously, namely whether there is more than one active ethnic group, past instances of civil war, and democratic institutions. We use both the existence of AMG (whether a country has AMG each year) and adoption of AMG (whether a country adopts AMG in a given year while excluding years where AMG is already present) as the dependent variable. Throughout the models, we rely on simple linear probability regressions, which in addition all include (1) time trends to account for the general rise in AMGs, (2) region-fixed effects to ensure that the association is not driven by one region, and (3) standard errors clustered by country, since error terms are likely correlated within a country over time.

**Table 1.** Correlates of AMGs

	DV: Existence of AMG				DV: Adoption of AMG			
	Ethnic Diversity	Civil War	Democratic institutions	Multivariate model	Ethnic Diversity	Civil War	Democratic institutions	Multivariate model
More than one active ethnic group	0*.*17***			0*.*06	0*.*01			−0*.*00
	(0*.*05)			(0*.*05)	(0*.*01)			(0*.*01)
Civil war in the past		0*.*23***		0*.*23***		0*.*03***		0*.*04***
		(0*.*04)		(0*.*04)		(0*.*01)		(0*.*01)
Polyarchy			0*.*94***	0*.*94***			0*.*20***	0*.*22***
			(0*.*23)	(0*.*20)			(0*.*06)	(0*.*05)
Year	0*.*01***	0*.*01***	0*.*01***	0*.*00***	0*.*00***	0*.*00***	0*.*00***	0*.*00
	(0*.*00)	(0*.*00)	(0*.*00)	(0*.*00)	(0*.*00)	(0*.*00)	(0*.*00)	(0*.*00)
Estimation method: Within region	Yes	Yes	Yes	Yes	Yes	Yes	Yes	Yes
Num. obs.	4647	4647	4579	4533	3665	3665	3572	3551
R^2^	0*.*13	0*.*18	0*.*18	0*.*25	0*.*01	0*.*02	0*.*03	0*.*04

***p < 0.01; **p < 0.05; *p < 0.1. Linear probability models with region-fixed effects and time trends. DV in models 1–4 is the existence of AMG, while DV in models 5–8 is adopting AMG (here, autocracies already with AMG are omitted). Time series from 1966 to 2020. Standard errors clustered by country in parentheses.

The first model (Ethnic Diversity) investigates how ethnic cleavages relate to AMG using a binary indicator of whether there are multiple active ethnic groups in a country, with data from the Ethnic Power Relations-dataset.^75^ We find that autocracies with multiple active ethnic groups are 17 percentage points more likely to have AMG in a given year compared to those with only one active ethnic group. They are also 1 percentage point more likely to adopt AMG, but this is not significant (*p* = 0.1). This indicates that ethnic diversity is better at explaining longevity of AMG governments than adoption. We provide further evidence of the association between ethnic diversity and AMG in Appendix H, where we use alternative measures of cultural and demographic cleavages.

A good example of this relationship is Malaysia, where the United Malays National Organization (UNMO) uninterruptedly dominated political life from independence in 1957 until the 2018 elections.^76^ The exclusively Malay party headed the Alliance Party (*Parti Perikatan*), which became the National Front (*Barisan Nasional*, or BN) coalition in 1973. The latter included several coalition partners, the main being the Malaysian-Chinese Association and the Malaysian Indian Congress (which were both part of the Alliance Party), the United Bumiputera Heritage Party (PBB), and formed an extended governmental coalition with the Sabah's People United Front (also known as Berjaya) and many other small opposition parties over the years – with each member retaining their own party labels both in elections and in government. Meanwhile, the main opposition parties – the Malaysian Islamic Party (PAS), the Chinese-dominated Democratic Action Party (DAP), and the multi-ethnic People's Justice Party (PKR) – were excluded from the ruling coalition and struggled to coalesce into an organized opposition front. As Pepinsky^77^ notes, it became clear over the years that “each opposition party [had] more in common with BN than it [did] with other opposition parties” – and yet they were unable to form a coalition because the ruling UNMO had selectively co-opted along ethnic lines the parties that could potentially have tipped the electoral scales in favour of the opposition.

In the second and sixth model of Table 1 (Civil War), we correlate AMG with whether a country has experienced a civil war in between 1946 and the given year, using the UCDP Armed Conflict Dataset.^78^ We find that autocracies with a history of intrastate violence are significantly more likely to adopt AMG. In fact, a country that has experienced civil war is 23 percentage points more likely to have multiple parties in government and 3 percentage points more likely to adopt AMG every year. In Appendix H, we show that the results are consistent regardless of how we operationalize past instances of civil war (for example, using the cumulative sum of all the years a country has experienced a civil war). This is in line with our theoretical expectations: We hypothesized that regimes experiencing civil war would be more likely to adopt multiparty governments as part of their conflict resolution effort. This may contribute to explaining why countries such as the Central African Republic or Rwanda have adopted multiparty governments while remaining autocratic.

A typical example of a setting where a civil war left a strong imprint on the system of nondemocratic government is Lebanon: The Taef agreement that was designed to end the two-decades-long war in 1989 specified a sectarian distribution of parliamentary seats and public office appointments.^79^ The latter were to be apportioned equally between Christian and Muslim political groups, when the pre-war ratio – which had been enshrined in the so-called “National Pact” gentlemen's agreement of 1943 – favoured Christians.^80^ An unwritten convention born at the end of the war further states that the president, who is the head of state and is elected by a two-thirds majority of the National Assembly, must be a Maronite Christian. He/she then consults the Speaker of the Assembly – a Shiia Muslim – and they invite a Sunni Muslim to form a cabinet as Prime Minister. The allocation of portfolios between cabinet members is then organized to reflect the sectarian balance. Because political parties in Lebanon – as in many other countries with stark religious divides – are based largely on sectarian adherence, this results in a government where portfolios are consistently split between parties, and there is power sharing between the different groups. This informal power-sharing arrangement is not static: It was reformed in 2008 with the Doha agreement.^81^ Under the new agreement, the opposition – Hezbollah at the time – was allocated enough cabinet positions to veto decisions made by the new cabinet of national unity. This gives further credence to our argument that power-sharing arrangements between different parties that are at a great distance ideologically can help sustain non-democratic systems of government, and that a history of civil conflict may facilitate the creation of such coalitions.^82^

In models 3 and 6 of Table 1 (Democratic Institutions), we correlate AGM with the Polyarchy index from V-Dem.^83^ We find that autocracies with some semblances of democratic institutions are more likely to both have and adopt multiparty governments, and this difference is statistically significant at the 5% level. Thus, even imperfect democratic institutions can work as a catalyst for power sharing across party lines in autocracies. This finding is robust to the use of alternative measures for democratic institutions which focus solely on the electoral component of democracy, as shown in Appendix H.

A good illustration is Zimbabwe, where the only time when power was shared between different parties in government was following a contested presidential election in 2008. Robert Mugabe's Zimbabwe African National Union-Patriotic Front (ZANU-PF) ruled as the only party in the game – and in government – from independence until 2008. Morgan Tsvangirai, the head of the Movement for Democratic Change (MDC-T), won the first round of the presidential election, but withdrew from the second round amidst threats of electoral violence. The opposition refused to acknowledge Mugabe's victory in the one-man second round, resulting in political deadlock. Both the international community and African leaders pushed for a power-sharing deal. Talks stalled for several weeks, because of mutual distrust, rivalry between the two factions of the MDC – led by Tsvangirai and by Arthur Mutambara respectively – and disagreements over the sharing of offices (in particular, the MDC initially refused to leave the highest executive office to Mugabe). After weeks of political stand-off and following the opposition's victory in the parliamentary elections held in August, an agreement was reached on 11 September 2008, and Tsvangirai became Prime Minister. While historical, the deal was largely political in nature.^84^ Some have stressed that the appointment of Tendai Biti of the MDC-T as Minister of Finance was a way for the ZANU-PF to dump the country’s dire economic situation on the former opposition party.^85^ Still, the existence of (largely imperfect) democratic institutions made AMG the only viable governing arrangement for both the ruling party and the co-opted opposition at the time.

At last, we include the three measures simultaneously in our analysis in columns 4 and 8 (Multivariate model). The measure of civil wars and democracy both remain positive and statistically significant, while the coefficient for ethnic diversity decreases in size and becomes statistically insignificant in both models. This suggests that ethnic heterogeneity may be a driver of AMG and explain longevity of AMGs, but that groups excluded from power either need a non-violent (democratic institutions) or a violent (civil war) way to gain access to the government, making these mediating variables.

In the Online Appendix, we present further analyses and robustness tests on the correlates of AMG. In Appendix, H we use alternative measures of social and cultural cleavages, civil war, and democracy to show that the results are consistent independent of the measure. We also refer to Appendix H for a longer discussion of how we may expect the various ways of measuring these three concepts to relate to our measure of multiparty government in dictatorships. Although the core of our theory is about the adoption of AMG, we also run the same analysis using three alternative measures as dependent variables: The number of parties, the share of cabinet positions, and the weighted Herfindahl index as the dependent variable. Results are presented in Appendix G, and our findings are generally comparable when we use these alternative outcome variables.

While the analysis provides strong evidence that multiparty government is a common feature of modern-day autocracies and correlates with socio-political factors in a meaningful way, this does not imply that coalition partners are granted any real influence in government.^86^ In the next section, we build new indicators to show that authoritarian coalition parties, at least to some degree, have tangible influence through their ministerial mandates.

## Do authoritarian coalition parties have any influence?

A concern is that the coalition parties included in our analysis could be completely subdued by the ruling party and that this may drive the findings. Yet, when we use the *v2elmulpar* from the V-Dem-project^87^ to only consider regimes where there either are no-parties or where all parties are de facto controlled by the dominant party, we find that there are only four AMGs in this group. These are Bulgaria (1966–1989), Poland (1966–1988), Algeria (1986–1989), and Rwanda (1992). Out of these only Bulgaria and Poland are true pro-forma AMGs. Moreover, it is extremely difficult to identify the partisan sub-units in regimes where the ruling party is in complete control, meaning that they generally are not covered in WhoGov. This for example concerns North Korea, where there are multiple parties, who collectively form the Democratic Front for the Reunification of Korea.

**Figure 5.**
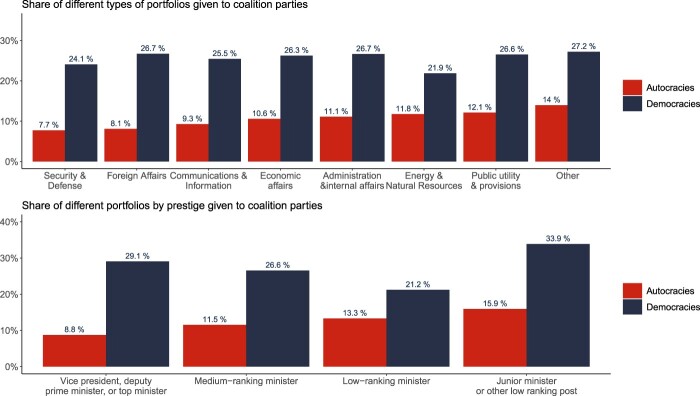
Share of positions given to coalition parties in democracies and autocracies, by portfolio and prestige.

Next, we shed further light on power sharing in AMGs by providing a descriptive analysis of the positions granted to coalition parties in democracies and autocracies respectively. The top panel in Figure 5 plots the share of the various cabinet portfolios given to parties other than that of the leader throughout the period 1966–2020. As expected, this share is higher in democracies than in democracies for all portfolios (21.9–27.2% in democracies *versus* 7.7–14.0% in autocracies). We also highlight some interesting variation across portfolios: The shares of Ministries of Security & Defense, Foreign Affairs, and Communications & Information granted to coalition parties respectively are the lowest in autocracies. This makes sense if one think of these portfolios as entailing access to strategic resources and responsibilities – such as control over the armed forces, access to diplomatic clout, and control of the government's information channels. Still, it is telling that on average, 7–8% of these ministries are controlled by individuals who do *not* belong to the ruler's party.

The bottom panel in Figure 5 classifies positions by importance rather than by portfolio. Unsurprisingly, autocracies allocate fewer of the most important positions to coalition partners compared to their democratic counterparts. Nevertheless, it is striking that almost 9% of the most powerful positions in government are given out to individuals from outside of the ruling party. Taken together, these descriptive results suggest that autocrats allocate positions of power within the government to outsiders to a higher extent than one would expect if there were no real power sharing across party lines. Still, these figures may conceal substantial variation in intra-governmental power-sharing dynamics, which we proceed to investigate through examples.

In many cases, even when power is shared between the ruling party and coalition parties, the allocation of cabinet posts helps maintain and entrench the dominance of the former. In Malaysia for instance, the ascendancy of the UNMO is reflected in the allocation of government posts: While the coalition parties taken together hold up to one-third of cabinet positions between 1966 and 2017, the posts are split between parties in such a way that no co-opted party holds more than four portfolios in any given year. Furthermore, the most important cabinet posts – Finance, Defense, Internal Security, Home Affairs, and Foreign Affairs – systematically go to UNMO members.^88^

Similarly, in Zimbabwe, a key point of contention following the 2008 presidential election was the allocation of cabinet posts, in particular the overall balance between parties, and control over the police and security forces. According to the final agreement, Mugabe would be President and chair of the cabinet, Tsvangirai the Prime Minister and head of the council of ministers in control of the implementation of government policies, and Mutambara deputy prime minister. The resulting government was made up of 19 ZANU-PF ministers, 15 MDC-T members, and 3 MDC-M ones. Importantly, Mugabe retained control of the defense and security forces as chair of the National Security Council.^89^ Bratton^90^ underscores the shortcomings of the “Global Political Agreement” stemming from the September 2008 talks, saying it was “externally driven, internally unbalanced, and short on incentives for at least one side to eschew violence.”

Overall, Figure 5 shows that real power sharing is less frequent in autocracies relative to democracies. Hence, while dictators are willing to enter coalitions with other parties to co-opt selected parts of the opposition and avoid threats to the regime, they rarely give away the most important posts. Yet, at the same time, we find substantial variation. In some cases, the leader must engage in real power-sharing agreements to survive in office. In Lebanon for instance, the country was only able to exit the political crisis it had been in for over 18 months through a new, formal agreement (the Doha agreement) specifying new power sharing terms which weakened the position of the leader's sectarian party, namely the Maronite Christians, to the benefit of the opposition Hezbollah. In particular, the new national unity cabinet that was agreed upon following a peace conference for national reconciliation arbitrated by the Arab League of Nations in June of 2008 specified that: “A Government of national unity will be formed with 30 ministers to be allocated as follows: 16 to the majority, 11 to the opposition, and three to the President.”^91^ *De facto*, this gave Hezbollah enough seats in cabinet to veto government initiatives, thus making for real power struggles within the government.

**Figure 6.**
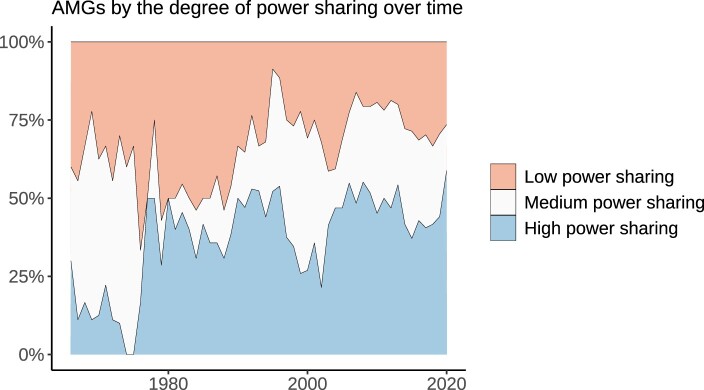
Proportion of AMGs by degree of power sharing. AMGs with high power sharing have a weighted Herfindahl-index between 0 and 0.5, those with medium have a score between 0.5 and 0.75, while those with low have a score between 0.75 and 1.

Lastly, we conduct a more fine-grained exploration of power distribution across AMGs over time in Figure 6. The figure displays the proportion of AMGs by degree of power sharing, using the weighted H-H index presented previously. Countries with an H-H index of 0.75–1 are classified as having a low degree of power sharing, those with between 0.5 and 0.75 have medium power sharing, while those with a score lower than 0.5 are classified as having a high degree of power sharing.

Interestingly, AMGs seem to engage in less power sharing during the period 1970–1990 than after the end of the Cold War. Crucially, we see that AMGs throughout the period display a higher degree of power sharing than what one would expect if they were solely *pro-forma* arrangements. Towards the end of the period, around half of all AMGs display signs of high power sharing across party lines, based on the number and importance of cabinet posts allocated to non-ruling parties. Hence it is not the case that coalition partners solely get few and minor cabinet portfolios. Instead, they typically get multiple, somewhat important positions associated with decision-making power over key mandates such as security or the economy.

Taken together, these analyses show that there are many cases where autocrats allocate positions of importance to members of coalition parties, thereby granting them at least partial control over certain policy areas. An important caveat is that the data does not allow us to investigate whether coalition partners are effectively able to influence policies and pressure the autocrat to deviate their preferences. In other words, we cannot directly relate the allocation of cabinet positions to economic, political, or diplomatic outcomes. Nevertheless, while we cannot be certain of what happens behind the closed doors of autocratic cabinets, the distribution of ministerial portfolios across regimes and over time provides strong evidence in favour of our argument that autocratic power sharing across party lines is not just for the show – instead, *bona fide* power sharing often occurs through strategic cross-party ministerial appointments.

## Conclusion and discussion on the rise of AMGs

This article builds on recent findings from the scholarship on authoritarianism to systematically study a phenomenon which have not been studied comparatively across a longer time period: Authoritarian multiparty governments. We present descriptive evidence on the increasing frequency of this phenomenon, show that these regimes are distinct from democracies, and demonstrate that this form of government does not emerge randomly among autocracies. Instead, it correlates with underlying social and historical cleavages, a history of civil wars, and quasi-democratic political institutions. Furthermore, fine-grained measures of power sharing in government suggest that coalition parties often yield real influence.

An open question is what fostered the rise of AMGs worldwide from the 1980s onward. We point to two concomitant global trends that are of relevance and based on our theory. First, after the end of the Cold War, civil conflicts become more frequent, but engaging in civil war has become increasingly expensive both for the autocrat and the opposition.^92^ Superpowers become more reluctant to intercede directly through militarized interventions and support, and, as a result, both rebel groups and incumbent governments have even less of an incentive to engage in conflict, because they will incur the associated cost in its entirety. This makes the adoption of multiparty government potentially more attractive to both sides, without necessarily achieving a full transition to democracy. That is not to say that civil wars necessarily become less frequent, but merely that in contexts where out-party government co-optation is an alternative, the benefits of this power-sharing strategy increase relative to its cost.

Second, authoritarian regimes are increasingly adopting democratic institutions such as parliaments and elections, partly due to the rising diplomatic and economic costs of being an “old-school” one-party state.^93^ While some particularly powerful or well-organized regimes like the People's Republic of China, or resource-rich states like Saudi Arabia have been able to withstand this process, many have not. The adoption of quasi-democratic institutions may trigger a process whereby opposition parties become increasingly threatening to the autocrat, causing the adoption of AMG to be the most palatable survival strategy.

Moreover, we know little about the nature of coalition parties in autocracies. While we discuss the different degrees of power sharing in AMGs, more data should be gathered on the coalition parties and their relationship with the ruling party. For example, future studies could gather data on their history or political views. Additionally, the article does not delve into the consequences of AMGs. As recent research has shown, ministerial cabinets are a key institution in authoritarian regimes, and their composition shapes the constraints that weigh on the autocrat.^94^ There is therefore good reason to think that the coalitional make-up of authoritarian governments ultimately bears implications for relevant policy and regime outcomes. These are avenues of research that are worth exploring, and our research aims at providing a useful analytical framework on which to build further empirical analyses of these questions.

Lastly, our findings relate to the literature on democratization, democratic backsliding, and how we think of “hybrid regimes.” In many autocracies, we look to the “democratic” opposition as the force that eventually will bring democracy to the country. However, this article shows that AMGs are alternative arrangements that benefit both the autocrat and select parts of the opposition, possibly making democratization less likely rather than more. Identifying this type of governing arrangement is a first step towards understanding its implications.

## Supplementary Material

Supplemental Material

## Notes

1 Hadenius and Teorell, “Pathways from Authoritarianism”; Magaloni et al. “Autocracies of the World”; Miller, “Autocratic Ruling Parties Dataset.”

2 Gerschewski, “Three Pillars of Stability”; Lust-Okar, *Structuring Conflict*; Reuter and Robertson, “Legislatures, Cooptation, and Protest.”

3 Blaydes, *Elections and Distributive Politics*; Geddes, “What Do We Know”; Slater, *Ordering Power*.

4 Magaloni, *Voting for Autocracy*, 200.

5 Boix and Svolik, “The Foundations”; Reuter, *The Origins of Dominant Parties*; Svolik, *The Politics of Authoritarian Rule*.

6 Kroeger, “Dominant Party Rule”; Meng, “Accessing the State.” We use the terms “political executive,” “ministerial cabinet,” “cabinet,” and “government” indifferently.

7 Arriola, Devaro, and Meng, “Democratic Subversion”; Meng, “Ruling Parties”; Van de Walle, “Tipping Games.”

8 Arriola, Devaro, and Meng, “Democratic Subversion”; Slater, “Party Cartelization, Indonesian-Style.”

9 Lust-Okar, *Structuring Conflict*.

10 Nyrup and Bramwell, “Who Governs?”

11 Blaydes, *Elections and Distributive Politics*, “What Do We Know”; Magaloni, *Voting for Autocracy*.

12 Huntington, *The Third Wave*.

13 Geddes, “What Do We Know.”

14 Levitsky and Way, *Competitive Authoritarianism*.

15 Hadenius and Teorell, “Pathways from Authoritarianism.”

16 Magaloni et al. “Autocracies of the World,” 8.

17 Diamond, “Thinking about Hybrid Regimes”; Andréas, “Elections without Democracy.”

18 Howard and Roessler, “Liberalizing Electoral Outcomes”; Levitsky and Way, *Competitive Authoritarianism*; Levitsky and Way, “The Rise.”

19 Geddes, Wright, and Frantz, “Autocratic Breakdown.”

20 Cox, “Authoritarian Elections and Leadership Succession”; Keefer, “Insurgency and Credible Commitment.”

21 Magaloni and Kricheli, “Political Order,” 125.

22 Diaz-Cayeros, Magaloni, and Weingast, “Tragic Brilliance”; Magaloni, *Voting for Autocracy*.

23 Blaydes, *Elections and Distributive Politics*; Gandhi, *Political Institutions under Dictatorship*.

24 Lust-Okar, *Structuring Conflict*, 1.

25 Ibid.

26 Kavasoglu, “Autocratic Ruling Parties”; Meng, “Ruling Parties.”

27 O’Donnel, “Modernization and Bureaucratic Authoritarianism,” 51.

28 Gandhi and Przeworski, “Cooperation, Cooptation, and Rebellion,” 91.

29 Frantz and Kendall-Taylor, “A Dictator’s Toolkit,” 335.

30 Malesky and Schuler, “Nodding or Needling.”

31 Reuter and Robertson, “Legislatures, Cooptation, and Protest.”

32 Meng, “Ruling Parties.”

33 Arriola, “Patronage and Political Stability”; Van de Walle, “Meet the New Boss.”

34 Gandhi and Przeworski, “Authoritarian Institutions,” 17.

35 Lust-Okar, “Elections under Authoritarianism”; Magaloni, *Voting for Autocracy*.

36 Arriola, Devaro, and Meng, “Democratic Subversion.”

37 Van de Walle, “Tipping Games.”

38 Arriola, Devaro, and Meng, “Democratic Subversion.”

39 Magaloni and Kricheli, “Political Order.”

40 Gandhi and Lust-Okar, “Elections under Authoritarianism.”

41 Magaloni and Kricheli, “Political Order”; Malesky and Schuler, “Nodding or Needling.”

42 Arriola, Devaro, and Meng, “Democratic Subversion.”

43 Arriola, “Patronage and Political Stability”; Gandhi and Przeworski, “Authoritarian Institutions”; Van de Walle, “Meet the New Boss.”

44 Arriola, Devaro, and Meng, “Democratic Subversion.”

45 Ibid., 10.

46 Bokobza et al., “The Morning After.”

47 Frantz and Kendall-Taylor, “A Dictator’s Toolkit,” 335.

48 Magaloni and Kricheli, “Political Order,” 126.

49 Lust-Okar, *Structuring Conflict*.

50 Meng, “Ruling Parties in Authoritarian Regimes.”

51 Arriola, Devaro, and Meng, “Democratic Subversion.”

52 Denny and Walter, “Ethnicity and Civil War”; Reynal-Querol, “Ethnicity, Political Systems.”

53 Ishiyama and Widmeier, “Territorial Control, Levels of Violence.”

54 Kovacs and Hatz, “Rebel-to-Party Transformations.”

55 Bunce and Wolchik, *Defeating Authoritarian Leaders*; Gandhi and Przeworski, “Cooperation, Cooptation, and Rebellion.”

56 Magaloni and Kricheli, “Political Order,” 126.

57 Bunce and Wolchik, *Defeating Authoritarian Leaders.*

58 Buckles, “Internal Opposition Dynamics.”

59 Jiménez, “Contesting Autocracy.”

60 Nyrup and Bramwell, “Who Governs?”

61 The article spurred additional data-gathering on the party variable and has led to corrections in the WhoGov dataset.

62 Nyrup and Bramwell, “Who Governs?”

63 We triangulate our results using The Database of Political Institutions 2020 (DPI) as an alternative data source in Appendix E. Contrary to WhoGov, DPI focuses on parliamentary alignment with the government and not on the government itself.

64 Boix, Miller, and Rosato, “A Complete Data Set.”

65 Cheibub, Gandhi, and Vreeland, “Democracy and Dictatorship Revisited.”

66 Ibid.

67 Teorell et al., “Measuring Polyarchy.”

68 Dahl, *Polyarchy*.

69 Hadenius and Teorell, “Pathways from Authoritarianism.”

70 Knutsen, Nygård, and Wig, “Autocratic Elections.”

71 Donno, “Elections and Democratization.”

72 Lust-Okar, *Structuring Conflict*.

73 We show the same figure for the year 1970 in Appendix F, and a table showing the list of AMGs for the years 1970, 1980, 1990, 2000, 2010, and 2020 in Appendix B.

74 As mentioned previously we, for comparability purposes, do not count independents as a separate party, but remove these throughout the analyses.

75 Vogt et al., “Integrating Data on Ethnicity.”

76 Pepinsky, “Malaysia.”

77 Ibid., 124.

78 Gleditsch et al., “Armed Conflict 1946–2001.”

79 Salamey, *The Government.*

80 Bogaards, “Counting Parties.”

81
https://www.refworld.org/docid/49913b61c.html

82 There were no parliamentary elections in Lebanon between 2009 and 2018.

83 Teorell et al., “Measuring Polyarchy.”

84 Bratton, *Power Politics in Zimbabwe*.

85 Godwin, *The Fear*.

86 Magaloni and Kricheli, “Political Order”; Malesky and Schuler, “Nodding or Needling.”

87 Pemstein et al., “The V-Dem Measurement Model.”

88 Pepinsky, “Malaysia.”

89 “Mugabe keeps the lion’s share”, *Business Day*, Johannesburg, September 13, 2008; “Details of power sharing deal revealed”, Agence France-Presse, September 15, 2008.

90 Bratton, *Power Politics in Zimbabwe*.

91 See https://www.securitycouncilreport.org/atf/cf/%7B65BFCF9B-6D27-4E9C-8CD3-CF6E4FF96FF9%7D/Lebanon%20S2008392.pdf

92 Kalyvas and Balcells, “International System.”

93 Howard and Roessler, “Liberalizing Electoral Outcomes”; Levitsky and Way, *Competitive Authoritarianism*.

94 Meng, “Accessing the State.”
