# Supplementary material for: Authoritarian multiparty governments
Source: Democratization. 2024 Apr 17;31(8):1669–94. doi: 10.1080/13510347.2024.2338858 (PMC11601049; doi:10.1080/13510347.2024.2338858)
Supplement: Supplemental Material [file FDEM_A_2338858_SM3775.pdf]

Appendix: For Online Publication

Contents

A Description of variables and coding decisions 2
B Autocracies with multiple parties in the government by decade 5
C Classification of importance 6
D Repeating the analysis with alternative measure of democracy 8
E Repeating the analysis with DPI 10
F Figure 3 for 1970 13
G Table 1 using alternative measures of AMG 14
H Table 1 using alternative measures of the independent variables 15
I Description of the coding of the party variable in WhoGov 18

A Description of variables and coding decisions

The analyses rely both on variables for measuring AMG and on background variables to investigate the correlates of AMG. The descriptive statistics for all variables are found in Table A1, while we discuss coding decisions and sources in the following sections. We exclude data on democracies from the descriptive statistics.

Table A1: Descriptive statistics

	Observations	Mean	Median	SD	Min	Max
Number of parties	4734	1.50	1.00	1.97	0.00	21.00
AMG (binary)	4734	0.24	0.00	0.43	0.00	1.00
Coalition parties' share of cabinet positions	4734	0.10	0.00	0.21	0.00	1.00
Weighted Herfindahl Index	3648	0.86	1.00	0.24	0.09	1.00
More than one active ethnic group	4647	0.85	1.00	0.35	0.00	1.00
Civil war in the past	4647	0.55	1.00	0.50	0.00	1.00
Polyarchy	4579	0.21	0.18	0.13	0.01	0.77

Variables for measuring AMG

Throughout the analyses we rely on several measures of AMG. In this appendix we provide further information on the coding decisions for the different variables.

Number of Parties We use the variable *n_party* from Nyrup and Bramwell (2020), which is a score for the number of parties represented in cabinet in a given year. The variable is calculated by counting the number of distinct parties amongst cabinet members using the *party* variable from the individual-level dataset. It should be noted that WhoGov only includes full-ranking ministers, deputy prime ministers, and the prime minister when counting the number of parties. Thus, junior ministers, presidents, and other people for whom WhoGov includes data, such as royals or advisors, are excluded. Cabinet members coded as independent and unknown are excluded.

Binary measure of AMG We code a binary measure of whether a country has AMG in a given year. The variable takes the score 1 if the variable *n_party* from WhoGov is 2 or larger, meaning that a country has more than one party represented in a given year. Thus, regimes with one party or zero parties in government gets the score 0.

Coalition Parties' Share of Cabinet Positions We calculate the share of cabinet members belonging to coalition parties in the government by creating a binary variable for each minister that shows whether a person belongs to the same party as the

leader or another party. Then we create a sum for both the total number of ministers and the number of ministers belonging to coalition parties, and find the share belonging to coalition parties. To identify the leader's party, we use the *leader* variable found in WhoGov. We include cabinet members coded as independent in count of total number of cabinet members, while those coded whose party is set to unknown are excluded. We include the same subset of cabinet members as for number of parties.

Weighted Herfindahl Index We construct a measure of concentration of power in government using a weighted Herfindahl–Hirschman Index (H-H), with more important posts receiving a higher weight, accounting for the fact that not all ministerial posts entail the same amount of prestige and responsibilities. The index ranges from 0 to 1, where 1 indicates that one party that controls all "points" - or all cabinet positions at once. By contrast, a low score on this scale indicates that many parties are controlling few posts each. Mathematically the index is constructed as $H = \sum_{i=1}^{N} m_i^2$ where m_i is the share of scores belonging to party m and N is the number of parties. Hence if there are two parties in government and they each have half of the cabinet positions, $H = 0.5^2 + 0.5^2 = 0.5$. To assign weights, we divide all cabinet positions into three categories; high, medium, and low prestige. The minister of defense, finance, foreign affairs, home/interior, the deputy prime minister, and, in presidential systems, the prime minister are categorized as high prestige. Ministries that controls significant resources, but has lesser status, are classified as medium prestige. These are, for example, agriculture, education, and transportation. At last, low-prestige positions are characterized by few resources and refers to ministries like youth, culture, and sports. We make some exceptions. For example, the minister of natural resources is considered of high prestige in OPEC countries. For more details on the coding, see Appendix C. If the same person controls multiple portfolios or ministries, we only take into account the most prestigious position. Then we assign a score of 3 to high prestige positions, while medium prestige positions are assigned a score of 2, while low prestige gets a score of 1. We then add all the scores together for each party. and use those to calculate the H-H index. We include the same subset of cabinet members as for number of parties.

Correlates of AMG

We correlate AMG with three factors that should be predictive of AMG, namely social and cultural cleavages, civil war, and democratic institutions. Below we discuss the main measures of these factors used in the paper.

More than one active ethnic group We create a binary variable based on on the variable actv_groups_count from the Ethnic Power Relations-dataset (Vogt et al. 2015) (or the GROWup-dataset (Girardin et al. 2015)). According to the codebook, an ethnic group is deemed active in a given group-year if it is currently physically present in a country and is not currently represented by an active ancestor or descendant.

A country-year with more than two or more active ethnic groups get the score 1, while country-years with only one active ethnic group gets the score 0.

Civil war in the past The measure indicates whether a country has experienced a civil war (intrastate war) prior to the given year. The measure starts in 1946, which is the earliest year with available data. We rely on data from the UCDP Armed Conflict Dataset (Gleditsch et al. 2002).

Polyarchy The v2x_polyarchy index. The index is formed by taking the average of, on the one hand, the sum of the indices measuring freedom of association (thick) (v2x_frassoc_thick), suffrage (v2x_suffr), clean elections (v2xel_frefair), elected executive (de jure) (v2x_accex) and freedom of expression (v2x_freexp_thick); and, on the other, the five-way interaction between those indices. Source: V-Dem (Teorell et al. 2019).

B Autocracies with multiple parties in the government by decade

In the table below we list all AMGs in WhoGov the first year of every decade and in 2016, which is the last year in WhoGov. We have proceeded to individual checks of the party affiliation for each minister in each of the country and years listed in the table.

	1970	Parties	1980	Parties	1990	Parties	2000	Parties	2010	Parties	2020	Parties
1	Indonesia	5	Malaysia	9	Malaysia	7	Malaysia	9	Iraq	16	Congo - Kinshasa	19
2	Lebanon	5	Morocco	5	Iran	6	Algeria	8	Sri Lanka	9	Niger	12
3	Malaysia	5	Poland	3	Uganda	5	Congo - Brazzaville	8	Chad	8	Malaysia	8
4	Bulgaria	2	Thailand	3	Bangladesh	4	Iran	7	Côte d'Ivoire	8	Mali	8
5	Laos	2	Bulgaria	2	Gabon	4	Morocco	5	Lebanon	8	Congo - Brazzaville	8
6	Morocco	2	Nicaragua	2	Lebanon	4	Uganda	5	Malaysia	8	Lebanon	7
7	Poland	2	Sri Lanka	2	Morocco	4	Fiji	4	Congo - Kinshasa	7	Benin	6
8	Tanzania	2	Suriname	2	Cambodia	3	Russia	4	Haiti	7	Pakistan	6
9					Comoros	3	Rwanda	4	Congo - Brazzaville	7	Thailand	6
10					Yemen	3	Cameroon	3	Morocco	6	Central African Republic	5
11					Senegal	2	Chad	3	Afghanistan	5	Afghanistan	4
12					Sri Lanka	2	Ecuador	3	Thailand	5	Guinea-Bissau	4
13							Gabon	3	Algeria	4	Iran	4
14							Lebanon	3	Central African Republic	4	Côte d'Ivoire	4
15							Liberia	3	Gabon	4	Maldives	4
16							Sierra Leone	3	Iran	4	Morocco	4
17							Angola	2	Kyrgyzstan	4	Uganda	4
18							Armenia	2	Niger	4	Yemen	4
19							Burundi	2	Sudan	4	Cameroon	3
20							Cambodia	2	Armenia	3	Equatorial Guinea	3
21							Congo - Kinshasa	2	Cameroon	3	Myanmar (Burma)	3
22							Georgia	2	Equatorial Guinea	3	South Sudan	3
23							Guinea-Bissau	2	Mauritania	3	Uzbekistan	3
24							Jordan	2	Rwanda	3	Algeria	2
25							Paraguay	2	Zimbabwe	3	Chad	2
26							Tajikistan	2	Cambodia	2	Comoros	2
27									Djibouti	2	Djibouti	2
28									Nigeria	2	Ethiopia	2
29									Togo	2	Gabon	2
30									Uzbekistan	2	Iraq	2
31									Yemen	2	Kyrgyzstan	2
32											Mauritania	2
33											Rwanda	2
34											Venezuela	2

Table B1: Autocracies with more than one party in government over time

C Classification of importance

Below we list the various portfolios existing in WhoGov, their classification in terms of importance, and their classification in terms of prestige. Following Krook and O'Brien (2012) we make two exceptions, namely 1) we consider any ministry having to do with natural resources, oil, or energy to be high prestige for OPEC members, and 2) we consider ministers of religious affairs to be a high-prestige positions in Iran, Afghanistan, Mauritania and Pakistan. We use use these to construct Figure 5 and the Weighted Herfindahl Index.

Table C1: Oversight over classification of importance

**Leader (1)**
Leader
**Prime minister/President (not leader) (2)**
President, chief of state, prime minister, who is not the leader
**Vice-president, deputy prime minister, and top minister (3)**
Vice-president, deputy prime minister, deputy chief of state
Full ranking minister of high prestige portfolio
**Medium-ranking minister (4)**
Full ranking minister of medium prestige portfolio
Attorney general, chief justice, or legal official
Governor (Military)
Member, royal family
Member, ruling group
**Low-ranking minister(5)**
Full-ranking minister of low prestige portfolio
Director of government agency
Government spokesperson
Governor (Regional)
**Junior minister or other low-ranking post (6)**
Junior minister (independent of prestige)
Advisor
Ambassador to the United States
Assistant advisor
Chief of staff
Deputy director of government agency
Governor (Central Bank)
Representative to the United Nations

[1]There can be several ministers in charge of, for example, defense. We only include the highest ranked minister as high prestige, while the rest are downgraded to medium prestige.

Table C2: List of Portfolios: Individual-level results

Portfolio	Prestige	Portfolio	Prestige
Defense, Military & National Security[1]	High	Foreign Relations[1]	High
Government, Interior & Home Affairs	High	Finance, Budget & Treasury[1]	High
Agriculture, Food, Fisheries & Livestock	Medium	Audit, Oversight & Internal Affairs	Medium
Civil Service	Medium	Communications & Information	Medium
Construction & Public Works	Medium	Correctional Services & Police	Medium
Culture & Heritage	Medium	Education, Training & Skills	Medium
Energy	Medium	Enterprises, Companies & Business	Medium
Environment	Medium	Executive & Legislative Relations	Medium
Foreign Economic Relations	Medium	General Economic Affairs	Medium
Health & Social Welfare	Medium	Housing	Medium
Industry & Commerce	Medium	Justice & Legal Affairs	Medium
Labor, Employment & Social Security	Medium	Local Government	Medium
Natural Resources	Medium	Planning & Development	Medium
Political Reform	Medium	Properties & Buildings	Medium
Religion	Medium	Regional	Medium
Tax, Revenue & Fiscal Policy	Medium	Transport	Medium
Ageing & Elderly	Low	Children & Family	Low
Immigration & Emigration	Low	Minorities	Low
Science, Technology & Research	Low	Sports	Low
Tourism	Low	Veterans	Low
Without Portfolio	Low	Women	Low
Youth	Low		
Other	Low		

D Repeating the analysis with alternative measure of democracy

In this appendix we replicate some of the main results using the DD index (Cheibub, Gandhi and Vreeland 2010) to show that our findings are independent of the choice of democracy indicator to distinguish democracies from autocracies.

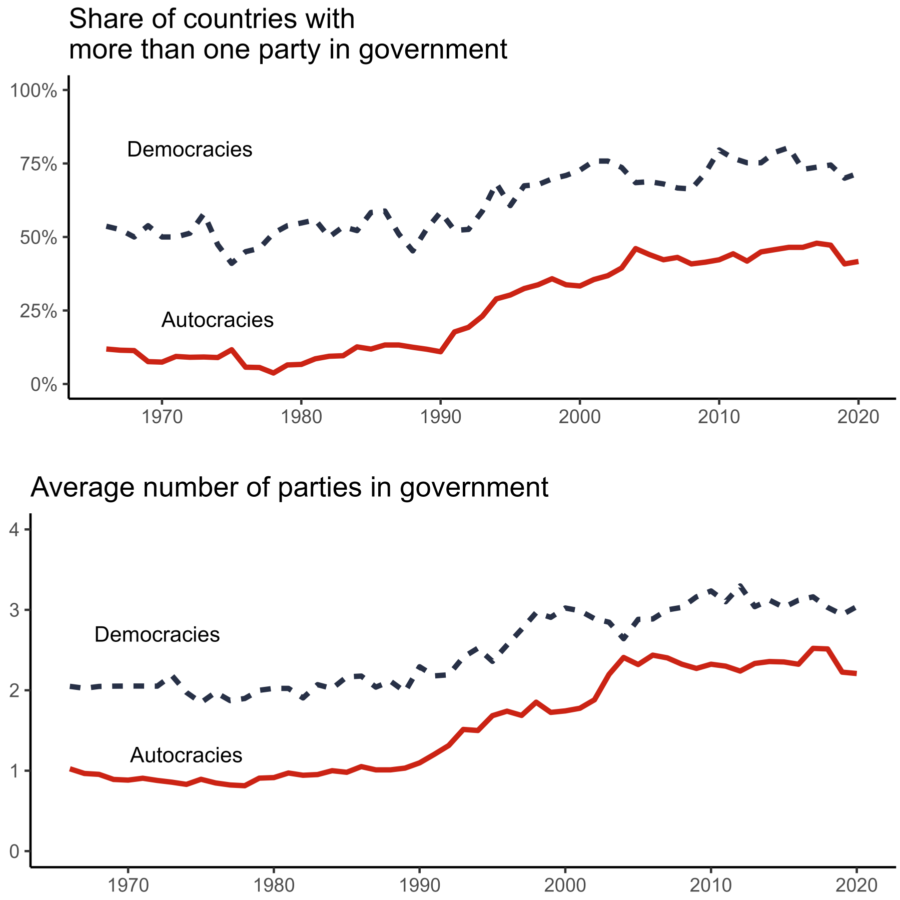

Figure D1: Evolution of (a) the number of parties in government and (b) the share of countries with more than one governing party. We use the DD index (Cheibub, Gandhi and Vreeland 2010) to measure democracy.

Table D1: Correlates of AMGs

	DV: Existence of AMG				DV: Adoption of AMG			
	Ethnic Diversity	Civil War	Democratic institutions	Multivariate model	Ethnic Diversity	Civil War	Democratic institutions	Multivariate model
More than one active ethnic group	0.17^{***}			0.07	0.01			0.00
	(0.04)			(0.05)	(0.01)			(0.01)
Civil war in the past		0.24^{***}		0.23^{***}		0.02^{***}		0.03^{***}
		(0.04)		(0.04)		(0.01)		(0.01)
Polyarchy			0.84^{***}	0.80^{***}			0.11^{***}	0.12^{***}
			(0.20)	(0.18)			(0.04)	(0.04)
Year	0.01^{***}	0.01^{***}	0.01^{***}	0.00^{***}	0.00^{***}	0.00^{***}	0.00^{***}	0.00^{**}
	(0.00)	(0.00)	(0.00)	(0.00)	(0.00)	(0.00)	(0.00)	(0.00)
Estimation method: Within region	Yes	Yes	Yes	Yes	Yes	Yes	Yes	Yes
Num. obs.	4582	4582	4528	4467	3644	3644	3553	3529
R^2	0.15	0.20	0.19	0.26	0.01	0.02	0.02	0.02

$^{***}p < 0.01$; $^{**}p < 0.05$; $^{*}p < 0.1$. Linear probability models with region fixed effects and time trends. DV in model 1-4 is existence of AMG, while DV in model 5-8 is adopting AMG (here, autocracies already with AMG are omitted). Time series from 1966-2020. Standard errors clustered by country in parentheses. We use DD-index to seperate democracies and autocracies.

E Repeating the analysis with DPI

In this appendix we replicate some of the main findings from the manuscript using the The Database of Political Institutions 2020 (DPI) (Cruz, Keefer and Scartascini 2020). DPI records information about parties represented in parliament that are aligned with the government. Thus, DPI focuses on parliamentary representation and not on the government itself. This is in contrast to WhoGov which focuses on the cabinet members. As a result, we do not expect the two measures to be perfectly consistent.

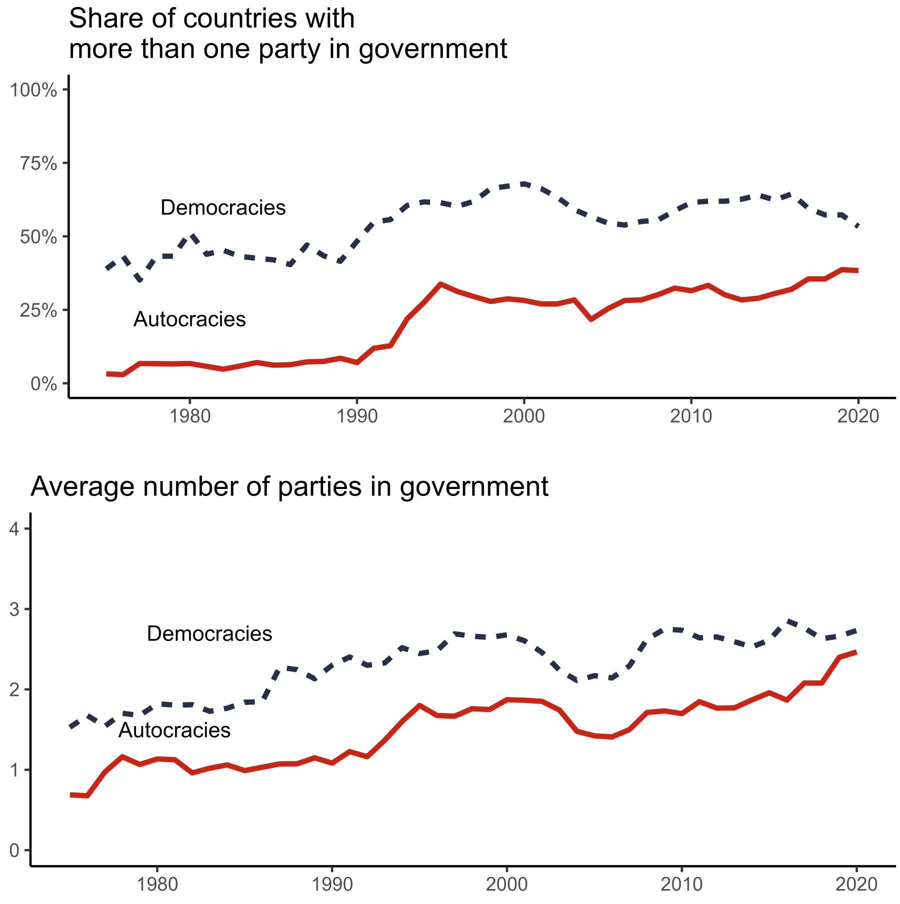

Figure E1: Evolution of (a) the number of parties in government and (b) the share of countries with more than one governing party. We use the data from the DPI (Cruz, Keefer and Scartascini 2020) to construct our measure.

Using DPI, we construct a measure of AMG, where a country is classified as AMG if there is more than one party listed as aligned with the government. Again, we rely on Boix, Miller and Rosato (2013) to distinguish democracies from autocracies. In figure E1 we replicate Figure 1, and find that we also see an increase in AMGs over time when using this alternative measure. This is the case both when looking at share of countries with AMG and at the average number of parties. Thus, this trend is not only found in WhoGov.

In addition, we correlate the measure of AMG from DPI with the three correlates of AMG in table E1. We find that polyarchy and past instances of civil war are significantly correlated with

AMG, both when looking at existence and adoption of AMG. Furthermore, the measure of social cleavages is positive and significant when looking at adoption, but insignificant when looking at existence of AMG. Again, it should be noted that DPI is distinct from WhoGov, causing us to not expect to fully replicate the results.

Table E1: Correlates of AMGs (Measure from DPI)

	DV: Existence of AMG				DV: Adoption of AMG			
	Ethnic Diversity	Civil War	Democratic institutions	Multivariate model	Ethnic Diversity	Civil War	Democratic institutions	Multivariate model
More than one active ethnic group	0.04			−0.02	0.02^{**}			0.00
	(0.08)			(0.07)	(0.01)			(0.01)
Civil war in the past		0.10^{**}		0.11^{***}		0.03^{***}		0.04^{***}
		(0.04)		(0.04)		(0.01)		(0.01)
Polyarchy			0.71^{***}	0.72^{***}			0.22^{***}	0.24^{***}
			(0.19)	(0.19)			(0.07)	(0.06)
Year	0.01^{***}	0.01^{***}	0.01^{***}	0.00^{***}	0.00^{***}	0.00^{***}	0.00	0.00
	(0.00)	(0.00)	(0.00)	(0.00)	(0.00)	(0.00)	(0.00)	(0.00)
Estimation method: Within region	Yes	Yes	Yes	Yes	Yes	Yes	Yes	Yes
Num. obs.	3805	3805	3752	3722	2902	2902	2824	2819
R^2	0.08	0.10	0.13	0.14	0.01	0.02	0.03	0.04

$^{***}p < 0.01$; $^{**}p < 0.05$; $^{*}p < 0.1$. Linear probability models with region fixed effects and time trends. DV in model 1-4 is existence of AMG measured using DPI, while DV in model 5-8 is adopting AMG measured using DPI (here, autocracies already with AMG are omitted). Time series from 1966-2020. Standard errors clustered by country in parentheses.

F Figure 3 for 1970

Figure F1 shows AMGs in 1970 in a similar way to figure 4.

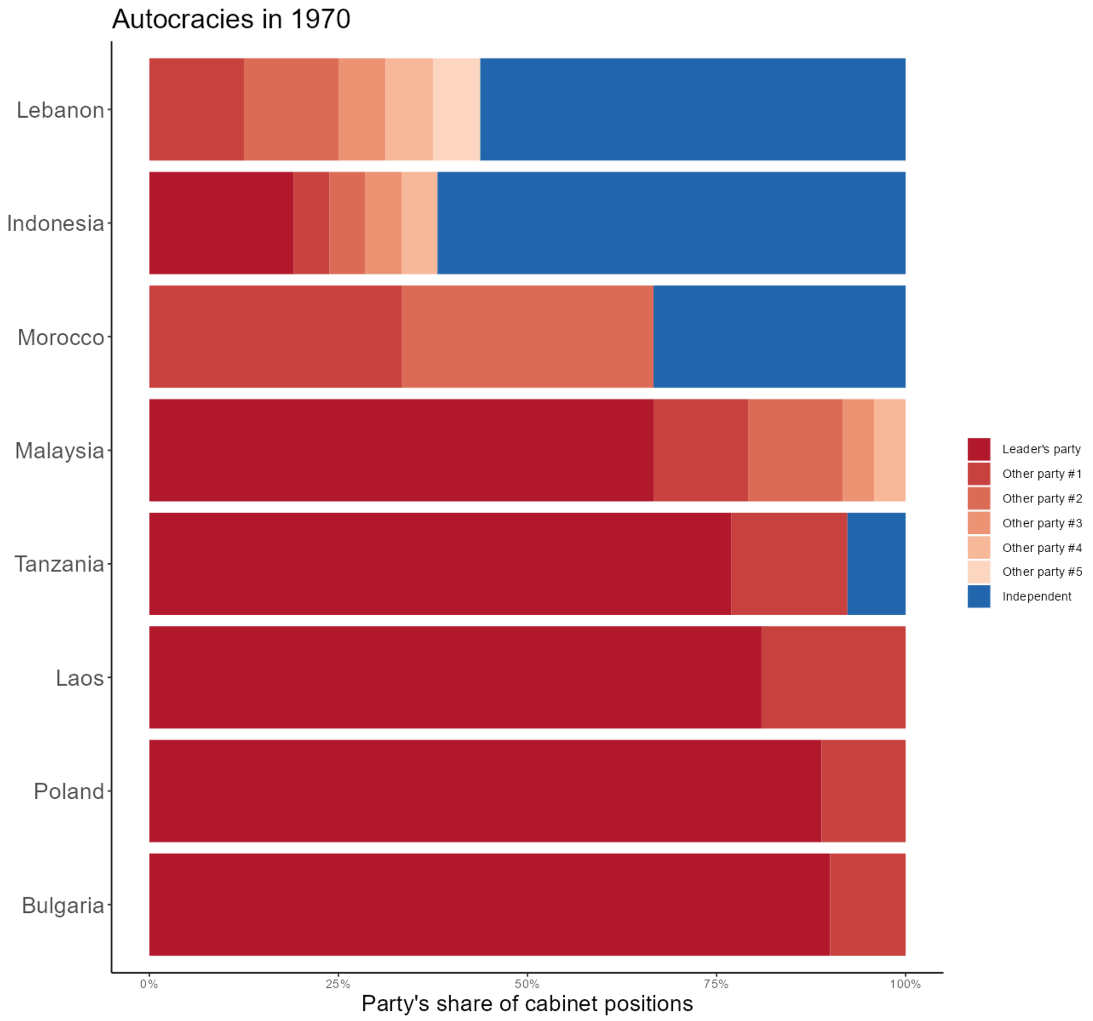

Figure F1: Distribution of cabinet positions by party in multiparty government autocracies ranked by the size of the largest party in 1970

G Table 1 using alternative measures of AMG

In the main text we correlate the three factors with the binary measure of AMG. However, as discussed in appendix A we also construct alternative measures of AMG. In this appendix we correlate these alternative measures with the measure of ethnic groups, past years with civil wars, and Polyarchy. We find many of the same patterns, and all coefficients are in the expected direction, although they are not all significant. Nonetheless, the theory focuses on whether countries adopt AMG; not the number of parties, coalition parties' share of cabinet positions nor the Weighted Herfindahl Index. Instead, we hope future research will look into predictors of these variables.

Table G1: Correlates of AMGs (number of parties)

	Ethnic Diversity	Civil War	Democratic institutions	Multivariate model
More than one active ethnic group	0.80 (0.24)***			0.36 (0.20)*
Civil war in the past		0.99 (0.22)***		0.96 (0.21)***
Polyarchy			4.46 (1.07)***	4.49 (0.94)***
Year	0.04 (0.01)***	0.03 (0.01)***	0.02 (0.01)***	0.02 (0.01)***
Estimation method: Within region	Yes	Yes	Yes	Yes
Num. obs.	4647	4647	4579	4533
R^2 (full model)	0.12	0.15	0.17	0.23
R^2 (proj model)	0.11	0.15	0.16	0.22

$^{***}p < 0.01$; $^{**}p < 0.05$; $^{*}p < 0.1$. OLS with region fixed effects and time trends. Dependent variable: Number of parties. Time series from 1966-2020. Standard errors clustered by country in parentheses.

Table G2: Correlates of AMGs (Coalition Parties' Share of Cabinet Positions)

	Ethnic Diversity	Civil War	Democratic institutions	Multivariate model
More than one active ethnic group	0.05 (0.02)**			0.01 (0.02)
Civil war in the past		0.08 (0.02)***		0.09 (0.02)***
Polyarchy			0.30 (0.09)***	0.30 (0.08)***
Year	0.00 (0.00)***	0.00 (0.00)***	0.00 (0.00)***	0.00 (0.00)***
Estimation method: Within region	Yes	Yes	Yes	Yes
Num. obs.	4647	4647	4579	4533
R^2 (full model)	0.08	0.11	0.10	0.14
R^2 (proj model)	0.07	0.10	0.09	0.13

$^{***}p < 0.01$; $^{**}p < 0.05$; $^{*}p < 0.1$. OLS with region fixed effects and time trends. Dependent variable: Coalition Parties' Share of Cabinet Positions. Time series from 1966-2020. Standard errors clustered by country in parentheses.

Table G3: Correlates of AMGs (Weighted Herfindahl Index)

	Ethnic Diversity	Civil War	Democratic institutions	Multivariate model
More than one active ethnic group	-0.05 (0.03)			-0.02 (0.04)
Civil war in the past		-0.11 (0.03)***		-0.13 (0.03)***
Polyarchy			-0.34 (0.16)**	-0.41 (0.14)***
Year	-0.01 (0.00)***	-0.00 (0.00)***	-0.00 (0.00)***	-0.00 (0.00)***
Estimation method: Within region	Yes	Yes	Yes	Yes
Num. obs.	3562	3562	3531	3486
R^2 (full model)	0.14	0.18	0.16	0.22
R^2 (proj model)	0.12	0.16	0.14	0.20

$^{***}p < 0.01$; $^{**}p < 0.05$; $^{*}p < 0.1$. Linear probability models with region fixed effects and time trends. Dependent variable: Weighted Herfindahl Index. Time series from 1966-2020. Standard errors clustered by country in parentheses.

H Table 1 using alternative measures of the independent variables

In this appendix, we present results using alternative measures of the three factors identified as correlates of existence of AMG and adoption of AMG in the main empirical specification. The results are reported in table H1 and H2.

In column 1-3 across both tables, we use three alternative measures of social and cultural cleavages. In column 1 we use Alesina et al. (2003) measure of linguistic fractionalization in the year 2000, while we in column 2 use ethnic fractionalization in the year 2000 from the same dataset. When looking at existence of AMG both measures are associated with a higher likelihood of a country having AMG, albeit only langauge fractionalization is statistically significant. In column 3 we use Fearon (2003)'s measure of cultural diversity, which combines ethnic and linguistic fractionalization. We find that culturally diverse autocracies are more likely to have a multiparty government, but that the coefficient is insignificant. When we instead look at adoption of AMG in table H2, we find that neither of the three measures are significant nor substantatively large. Again, this indicates that fractionalization may help explain longevity of AMGs but is a poor predictor of adoption of AMGs.

Next, in column 4-6, we employ three alternative measures of civil war. In column 4, we use a binary measure of whether there is an ongoing civil war in a country using (Gleditsch et al. 2002), and find that countries with AMG are much more likely to experience a civil war. Furthermore, in column 5, we use the number of past years with civil war, again based on (Gleditsch et al. 2002), and find that countries with more years of past civil war are more likely to have and adopt AMGs. Furthermore, in column 6 we the Major Episodes of Political Violence from the Center for Systemic Peace (Marshall 2019) to measure whether a country has a civil war in the past, and find that this measure also positively predicts AMG.

At last, we use three alternative measures of democratic institutions in column 7-9. In column 7 we use a continuous measure of democracy, the Polity IV score (Marshall, Gurr and Jaggers 2019), and find that the coefficient is also positive and significant. This index captures the level of authortarianism on a scale ranging from -10 (hereditary monarchy) to +10 (consolidated democracy). Specifically, the Polity score captures the quality of executive recruitment, constrains on the executive, and political competition. It differs from the V-Dem polyarchy index in its method of aggregation, and in the fact that it is less conservative – in particular its suffrage component is less

demanding (Teorell et al. 2016).

Likewise, in column 7 and 8, we use the core civil society index and the electoral component index which both are from V-Dem (Coppedge et al. 2020), and find that both are positive and significant. The CCSI index captures the robustness of civil society, and results thus show that a stronger realm of social contestation with the dictatorial regime is positively associated with more parties being included in government. The electoral component index captures, in addition to how freely political and civil society organizations operate, the extent of suffrage, the degree of freedom and fairness of elections, and mode of selection of the chief executive. Taken together, these continuous measures of democracy are useful theoretical complements to the Polyarchy index used in the main specification.

Overall, we therefore find that AMG also correlates positively and significantly with these alternative measures, strengthening our confidence that the three correlates of AMG identified in the main analysis are in fact relevant. However, ethnic divides may be better at explaining the longeivity of AMG and degree of power-sharing than the adoption of AMG.

Table H1: Correlates of AMGs

	(1)	(2)	(3)	(4)	(5)	(6)	(7)	(8)	(9)
Language Fractionalization (Alesina et. al)	0.16^{*}								
	(0.09)								
Linguistic Fractionalization (Alesina et. al)		0.06							
		(0.11)							
Cultural diversity (Fearon)			0.16						
			(0.13)						
Civil war (in the given year)				0.22^{***}					
				(0.05)					
Past years with civil war					0.01^{**}				
					(0.00)				
Civil war in the past (CSP)						0.19^{***}			
						(0.06)			
Polity IV							0.03^{***}		
							(0.01)		
Core civil society index								0.46^{***}	
								(0.10)	
Electoral component index									0.60^{***}
									(0.11)
Year	0.01^{***}	0.01^{***}	0.01^{***}	0.01^{***}	0.01^{***}	0.01^{***}	0.01^{***}	0.01^{***}	0.01^{***}
	(0.00)	(0.00)	(0.00)	(0.00)	(0.00)	(0.00)	(0.00)	(0.00)	(0.00)
Estimation method: Within region	Yes	Yes	Yes	Yes	Yes	Yes	Yes	Yes	Yes
Num. obs.	4307	4439	4294	4647	4647	4466	4381	4579	4579
R^2 (full model)	0.11	0.11	0.12	0.16	0.15	0.15	0.21	0.17	0.22
R^2 (proj model)	0.10	0.10	0.11	0.15	0.15	0.15	0.21	0.17	0.22

$^{***}p < 0.01$; $^{**}p < 0.05$; $^{*}p < 0.1$. Linear probability models with region fixed effects and time trends. Dependent variable: Existence of Authoritarian Multiparty Government. Time series from 1966-2020. Standard errors clustered by country in parentheses.

Table H2: Correlates of adopting AMG

	(1)	(2)	(3)	(4)	(5)	(6)	(7)	(8)	(9)
Language Fractionalization (Alesina et. al)	0.00								
	(0.01)								
Linguistic Fractionalization (Alesina et. al)		−0.00							
		(0.02)							
Cultural diversity (Fearon)			0.00						
			(0.02)						
Civil war (in the given year)				0.04^{***}					
				(0.01)					
Past years with civil war					0.00^{*}				
					(0.00)				
Civil war in the past (CSP)						0.03^{**}			
						(0.01)			
Polity IV							0.01^{***}		
							(0.00)		
Core civil society index								0.10^{***}	
								(0.02)	
Electoral component index									0.13^{***}
									(0.03)
Year	0.00^{***}	0.00^{***}	0.00^{***}	0.00^{***}	0.00^{***}	0.00^{***}	0.00^{***}	0.00^{***}	0.00^{**}
	(0.00)	(0.00)	(0.00)	(0.00)	(0.00)	(0.00)	(0.00)	(0.00)	(0.00)
Estimation method: Within region	Yes	Yes	Yes	Yes	Yes	Yes	Yes	Yes	Yes
Num. obs.	3376	3498	3349	3665	3665	3531	3480	3572	3572
R^2 (full model)	0.01	0.01	0.01	0.02	0.01	0.02	0.04	0.03	0.04
R^2 (proj model)	0.01	0.01	0.01	0.02	0.01	0.02	0.04	0.03	0.04

$^{***}p < 0.01$; $^{**}p < 0.05$; $^{*}p < 0.1$. Linear probability models with region fixed effects and time trends. Dependent variable: Adoption of Authoritarian Multiparty Government. Time series from 1966-2020. Standard errors clustered by country in parentheses.

I Description of the coding of the party variable in WhoGov

The party variable was coded in several steps. First, extensive historical accounts of each country and existing databases were explored to determine whether there was more than one party in government at any point – including the *Political Handbook of the World*, Miller (2020), the DPI (Beck et al. 2001), and Cheibub, Gandhi and Vreeland (2010) updated by Bjørnskov and Rode (2018). In some cases, it is easy to rule out the existence of multiparty governments. Some autocracies, such as Saudi Arabia and Swaziland, have a blanket ban on political parties. In other cases, one can safely assume that all members of the government belonged to the same party. For instance, Zaire (1967-1992) mandated that all adult citizens were members of the ruling party.

For the remaining cases, each minister's party label was coded individually, using thousands of sources.[9] A number of coding decisions are of relevance for this article. First, when deciding what counts as party affiliation, *WhoGov* aims to code only "card carrying" members of the party, such as those who run for office on behalf of the party. Often, politicians will have a relationship with, openly sympathize with, or be nominated by a party, all the while not being member of said party. Second, parties often form electoral coalitions gathering several parties. *WhoGov* codes politicians as belonging to the smallest possible political unit. Third, in some countries, parties exist but play a minor role, so that party affiliation is often absent from ministers' biographical information. Similarly, in a handful of countries, data is very sparse, but historical documents confirm that multiple parties are often represented in government. In these cases, the party affiliation could not be recorded for all ministers with sufficient certainty. Fourth, the coding of party affiliation is time-variant, taking into account cases when members of the government switch to a different party.

The end product is a list of members of the cabinet, including their party affiliation, for all years in the period 1966-2021, in any given country. However, due to limited availability on other variables we only use the data up to 2020 throughout the analyses. By focusing on individual cabinet members, we are better able to detect when and which coalition parties are included in the country's political system at the highest level of decision-making, than by looking at the legalization of parties or focusing on elections. Yet, we acknowledge that there may be imprecise data points in cases where there was insufficient historical information.[10] That being said, we are confident that *WhoGov* presents the best existing data to examine the rise of multiparty governments in autocracies. To further strengthen the confidence in our results, we conducted an extensive double-checking of the partisan composition of all governments classified as AMGs in 1970, 1980, 1990, 2000, 2010 and 2016 respectively.

---

[9] For most countries, the primary sources are the *Political Handbook of the World*, *Europa Regional Surveys of the World*, Bértoa (2020), The Presidential Cabinets Project (Camerlo and Martinez-Gallardo 2020), Wikileaks, LinkedIn, Historical Dictionaries for various countries, and Wikipedia. For a detailed, country-by-country description of the coding process, we kindly refer the reader to Appendix F of Nyrup and Bramwell (2020).

[10] In total, party affiliation is coded for 94 percent of all cabinet members.

References for the appendix

Alesina, Alberto, Arnaud Devleeschauwer, William Easterly, Sergio Kurlat and Romain Wacziarg. 2003. "Fractionalization." *Journal of Economic growth* 8(2):155–194.

Beck, Thorsten, George Clarke, Alberto Groff, Philip Keefer and Patrick Walsh. 2001. "New tools in comparative political economy: The database of political institutions." *The World Bank Economic Review* 15(1):165–176.

Bértoa, Fernando Casal. 2020. "Database on WHO GOVERNS in Europe and beyond, PSGo." Available at https://whogoverns.eu/ (24/02/2020).

Bjørnskov, Christian and Martin Rode. 2018. "Regime Types and Regime Change: A New Dataset.".

Boix, Carles, Michael Miller and Sebastian Rosato. 2013. "A complete data set of political regimes, 1800–2007." *Comparative Political Studies* 46(12):1523–1554.

Camerlo, Marcelo and Cecilia Martinez-Gallardo. 2020. "The Presidential Cabinets Project." Available at https://www.presidentialcabinets.org/ (24/02/2020).

Cheibub, José Antonio, Jennifer Gandhi and James Raymond Vreeland. 2010. "Democracy and dictatorship revisited." *Public Choice* 143(1-2):67–101.

Coppedge, Michael, John Gerring, Carl Henrik Knutsen, Staffan I. Lindberg, Jan Teorell, David Altman, Michael Bernhard, M. Steven Fish, Adam Glynn, Allen Hicken, Anna L uhrmann, Kyle L. Marquardt, Pamela Paxton, Kelly McMann, Daniel Pemstein, Brigitte Seim, Rachel Sigman, Svend-Erik Skaaning, Jeffrey Staton, Steven Wilson, Agnes Cornell, Nazifa Alizada, Lisa Gastaldi, Haakon Gjerløw, Garry Hindle, Nina Ilchenko, Laura Maxwell, Valeriya Mechkova, Juraj Medzihorsky, Johannes von R omer, Aksel Sundstr om, Eitan Tzelgov, Yi ting Wang, Tore Wig and Daniel Zilblatt. 2020. "V-Dem Country-Year/Country-Date Dataset v10.".
URL: *https://www.v-dem.net/en/data/data-version-10/*

Cruz, Cesi, Philip Keefer and Carlos Scartascini. 2020. "The Database of Political Institutions 2020 (DPI 2020)." *Washington, Banco Interamericano de Desarrollo* .

Fearon, James D. 2003. "Ethnic and cultural diversity by country." *Journal of economic growth* 8(2):195–222.

Gleditsch, Nils Petter, Peter Wallensteen, Mikael Eriksson, Margareta Sollenberg and Håvard Strand. 2002. "Armed conflict 1946-2001: A new dataset." *Journal of peace research* 39(5):615–637.

Krook, Mona Lena and Diana Z O'Brien. 2012. "All the president's men? The appointment of female cabinet ministers worldwide." *The Journal of Politics* 74(3):840–855.

Marshall, Monty G. 2019. "Major episodes of political violence (MEPV) and conflict regions, 1946–2008." *Center for Systemic Peace* .

Marshall, Monty G, Ted Robert Gurr and Keith Jaggers. 2019. “Polity IV project: Political regime characteristics and transitions, 1800-2018.” *Center for Systemic Peace* .

Miller, Michael K. 2020. “The autocratic ruling parties dataset: Origins, durability, and death.” *Journal of Conflict Resolution* 64(4):756–782.

Nyrup, Jacob and Stuart Bramwell. 2020. “Who Governs? A New Global Dataset on Members of Cabinets.” *American Political Science Review* 114(4):1366–1374.

Teorell, Jan, Michael Coppedge, Staffan Lindberg and Svend-Erik Skaaning. 2019. “Measuring polyarchy across the globe, 1900–2017.” *Studies in Comparative International Development* 54(1):71–95.

Teorell, Jan, Michael Coppedge, Svend-Erik Skaaning and Staffan I. Lindberg. 2016. “Measuring Electoral Democracy with V-Dem Data: Introducing a New Polyarchy Index.” *SSRN Electronic Journal* .

Vogt, Manuel, Nils-Christian Bormann, Seraina Rüegger, Lars-Erik Cederman, Philipp Hunziker and Luc Girardin. 2015. “Integrating data on ethnicity, geography, and conflict: The ethnic power relations data set family.” *Journal of Conflict Resolution* 59(7):1327–1342.
